# Supplementary material for: Aqueous synthesis of highly functional, hydrophobic, and chemically recyclable cellulose nanomaterials through oxime ligation
Source: Nat Commun. 2022 Nov 14;13:6924. doi: 10.1038/s41467-022-34697-5 (PMC9663568; doi:10.1038/s41467-022-34697-5)
Supplement: Supplementary file 1 — Supplementary Information [file 41467_2022_34697_MOESM1_ESM.pdf]

Supplementary Materials for

Aqueous synthesis of highly functional, hydrophobic, and chemically recyclable cellulose nanomaterials through oxime ligation

Elena Subbotina*, Farsa Ram, Sergey V. Dvinskikh, Lars A. Berglund, Peter Olsén*

*Corresponding author. Email: elenasu@kth.se, polsen@kth.se

Table of Content

This PDF file includes:

Supplementary Methods
Supplementary Figs. 1 to 37
Tables 1 to 3
Supplementary References (1 to 4)

Supplementary methods

Synthesis of starting materials

Preparation of O-substituted hydroxyl amines

Synthesis of O-allylhydroxylamine(8) and O-propargylhydroxylamine hydrochlorides (9)

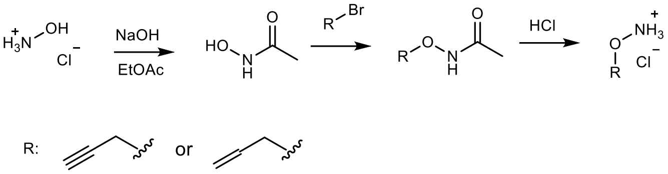

O-allylhydroxylamine and O-propargylhydroxylamine hydrochlorides were prepared following modified literature procedure. (*1*) Hydroxylamine hydrochloride (1.1 g, 15.9 mmol) was dissolved in 16 mL EtOAc/H_2O (1/2, v/v) mixture, containing NaOH (1.36 g, 34 mmol). The solution was stirred at 0 °C for 4 hours. Allyl bromide (1.2 mL, 13.9 mmol) or propargyl bromide (1.2 mL, 13.9 mmol) was added and the reaction mixture was stirred at 50 °C for several hours. The reaction mixture was extracted with EtOAc/H_2O. The combined organic phase was collected and dried over anhydrous Na_2SO_4. The solvent was removed under reduced pressure using rotary evaporator. The obtained oil was dissolved in EtOH (100 mL) and 0.150 mL of HCl (conc.) was added to the solution. The solution was stirred at 65 °C for 6 hours. Upon the completion of the reaction solvent was removed under reduced pressure using rotary evaporator to give the product as yellowish solid.
O-Allylhydroxylamine hydrochloride (**8**):
NMR data match previously reported. (*2*)
O-Propargylhydroxylamine hydrochloride (**9**):
^{1}H NMR (400 MHz, D_2O) δ 4.74 (d, J = 2.3 Hz, 2H), 3.16 (t, J = 2.3 Hz, 1H).

^{13}C NMR (101 MHz, D_2O) δ 80.1, 74.9, 62.6.

Synthesis of 1,2-di-*p*-tolyldiazene

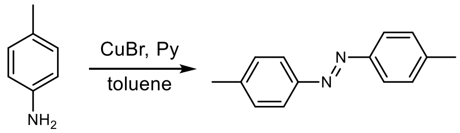

1,2-di-p-tolyldiazene was prepared following a procedure described in literature.(*3*) p-toluidine (5g, 46.7 mmol), CuBr (93 mg, 0.65 mmol) and pyridine (150 mg, 1.9 mmol) were were placed in a round bottom flask, 30 mL of toluene was added to the flask. The reaction mixture was stirred for 16 hours at 60 °C. Upon the completion of the reaction, the content of the flask was concentrated at reduced pressure. The product was purified through column chromatography on silica gel, using hexane as an eluent.
NMR data match previously reported.(*3*)

Synthesis of 1,2-bis(4-(bromomethyl)phenyl)diazene

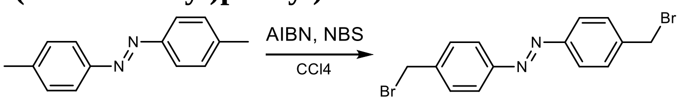

1,2-bis(4-(bromomethyl)phenyl)diazene was prepared following a procedure described in literature. (*4*) 1,2-bis(4-(bromomethyl)phenyl)diazene (2.2 g, 10.3 mmol) and was placed in round

bottom flask followed by an addition of CCl_4 (40 mL), N-bromosuccinimide (4.2 g, 23.7 mmol) and azobisisobutyronitrile (130 mg, 0.77 mmol). The mixture was stirred overnight at 70 °C. The product was filtered, washed with chloroform and water, and dried under reduced pressure to give the final product.
NMR data match previously reported.(*4*)

General procedure for preparation O-substitutedhydroxylamine hydrotriflates

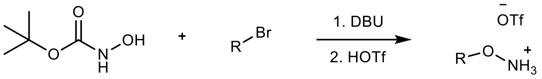

Boc-protected hydroxylamine (1.05 equiv., for bifunctional bromides 2.1 equiv. were used), corresponding bromide (1 equiv.), 1,8-Diazabicyclo(5.4.0)undec-7-ene (DBU) (1.05 equiv., for bifunctional bromides 2.1 equiv. were used) were dissolved in DCM. The reaction mixture was stirred at room temperature for several hours. The progress of the reaction was monitored by NMR. Upon the completion the reaction mixture was extracted with water. The combined organic phase was collected and dried over anhydrous Na_2SO_4. The obtained DCM solution was cooled to 0 °C. Triflic acid (1 equiv.) was added to the solution dropwise. The reaction mixture was left to stir for an hour. The reaction was accompanied by an evolution of gas and formation of precipitate. The final product was collected by vacuum filtration, washed with cold DCM and dried under reduced pressure.

O-(*p*-Trifluoromethylbenzyl)hydroxylamine hydrotriflate (3):

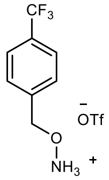

^{1}H NMR (400 MHz, D_2O) δ 7.77 (m, 2H), 7.62 (m, 2H), 5.15 (s, 2H).
^{13}C NMR (101 MHz, DMSO) δ 138.6 (br), 130.0 (br), 126.0 (br), 123.2 (br), 121.1 (q, JC-F = 324.7 Hz), 75.4.

O-(*p*-Nitrobenzyl)hydroxylamine hydrotriflate (2):

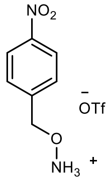

^{1}H NMR (400 MHz, D_2O) δ 8.24 (d, J = 8.8 Hz, 2H), 7.64 (d, J = 8.8 Hz, 2H), 5.18 (s, 2H).
^{13}C NMR (101 MHz, D_2O) δ 148.1, 140.0, 129.7, 123.95, 119.5 (q, $J_{C\text{-}F}$ = 317.3 Hz), 75.4.

Pentafluorobenzylhydroxylamine hydrotriflate (4):

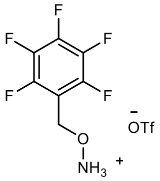

^{1}H NMR (400 MHz, D_2O) δ 5.19 (s, 2H).
^{13}C NMR (101 MHz, D_2O) δ 145.87 (m, J^{1}_{C-F} = 253.9 Hz), 142.40 (m, J^{1}_{C-F} = 260.0 Hz), 137.39 (m, J^{1}_{C-F} = 247.3 Hz), 119.5 (q, J_{C-F} = 317.3 Hz), 106.39 (m), 63.50.

4-(2-(aminooxy)ethyl)phenol hydrotriflate (5):

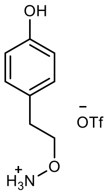

^{1}H NMR (400 MHz, D_2O) δ 7.15 (d, J = 8.5 Hz, 2H), 6.82 (d, J = 8.5 Hz, 2H), 4.21 (t, J = 6.5 Hz, 2H), 2.88 (t, J = 6.5 Hz, 2H).
^{13}C NMR (101 MHz, D_2O) δ 154.1, 130.1, 129.0, 119.5 (q, J_{C-F} = 317.3 Hz), 115.4, 75.7, 32.4.

4,4'-diaminoxymethylazobenzene hydrotriflate (10):

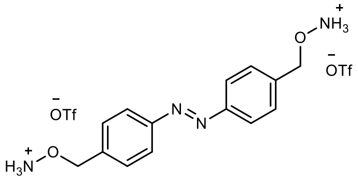

^{1}H NMR (400 MHz, D_2O) δ 7.74 (d, J = 8.4 Hz, 2H), 7.55 (d, J = 8.4 Hz, 2H), 5.08 (s, 2H).
^{13}C NMR (101 MHz, D_2O) δ 152.4, 135.9, 130.3, 122.9, 119.5 (q, JC-F = 317.3 Hz), 76.2.

O,O'-bis-(1,5-dihydroxylamine)pentane hydrotriflate (6) :

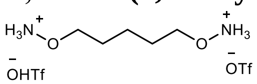

The reaction was performed in THF at 80 °C. The progress of the reaction was monitored by NMR. An additional amount of DBU (ca. 0.3 equiv.) was added into the reaction mixture after ca. 6 hours. The final product (1,5-dihydroxylaminepentane hydrotriflate) did not form precipitate upon the addition of triflic acid. The solvent was removed under reduced pressure and the flask containing the product was left under vacuum at room temperature overnight. The developed white crystals were collected and washed with hexane.

^{1}H NMR (400 MHz, D_2O) δ 4.06 (t, J = 6.4 Hz, 4H), 1.70 (s, 4H), 1.46 (s, 2H).

^{13}C NMR (101 MHz, D_2O) δ 75.2, 26.5, 21.0.

Determination of aldehyde content of DA-CNF (DO)

A piece of wet cake (ca. 100-200 mg on dry basis) was paced into 25 mL solution of hydroxylamine hydrochloride (400 mg) adjusted to pH 4 with NaOH. The solution was stirred for 4 hours. The obtained solution was titrated back to pH of 4 using NaOH solution. DA-CNF was removed form the resultant solution via vacuum filtration, washed with distilled water and dried under vacuum. Aldehyde content, degree of oxidation (DO, mol/g) of DA-CNF wet cakes was estimated as follows (as an average of three repetitions):

$$DO\ (\frac{\mathrm{mol}}{\mathrm{g}}) = \frac{m_{solution} \times \omega_{NaOH}}{M_{w(NaOH)} \times m_{DA-CNF}}\ (1)$$

Where $\mathrm{m_{solution}}$ is a mass of the added solution of NaOH (g), $\omega(_{\mathrm{NaOH})}$ is a weight fraction of NaOH in solution (%), $\mathrm{M_{w(NaOH)}}$ is molecular weight of NaOH (40 g/mol), $\mathrm{m_{DA-CNF}}$ is dry mass of DA-CNF (g).

Characterization of DA-CNF and R-ON-CNF

Determination of degree of functionalization (DF) gravimetrically

First, a dry content of the corresponding DA-CNF wet cake used for the preparation of R-ON-CNF was measured by weighting a piece of the wet cake before and after drying under vacuum at 40 °C for 12 hours as follows:

$$Dry\ content, \% = \frac{m_{DA-CNF\,dry}}{m_{DA-CNF\,wet}}\ (2)$$

A DA-CNF wet cake used for the preparation of R-ON-CNF and the corresponding dry R-ON-CNF film prepared as described in section 3.4 were weighted. DF was calculated as follows:

$$DF(\frac{\mathrm{mol}}{\mathrm{g}}) = \frac{(m_{R-ON-CNF\,dry} - m_{DA-CNF\,wet} \times Dry\ content)/(Mw_{R-ONH_2} - Mw_{H_2O})}{m_{DA-CNF\,wet} \times Dry\ content}\ (3)$$

Where $\mathrm{m_{R-ON-CNF\,dry}}$ (g) is a weight of the dry R-ON-CNF film after the reaction, $\mathrm{m_{DA-CNF\,wet}}$ (g) is a weight of the wet cake prior to the reaction. $\mathrm{Mw_{R-ONH_2}}$ (g/mol) is Mw of the corresponding O-substituted hydroxylamine hydrochloride, and $\mathrm{Mw_{H_2O}}$ (18 g/mol) is a molecular weight of water (by-product of the reaction).

Determination of degree of functionalization (DF) using ^{13}C CP/MAS NMR

First, average Mw of anhydroglucose unit (AGU) of DA-CNF ($\mathrm{AGU_{DA\text{-}CNF}}$) used for the preparation of the corresponding R-ON-CNF was calculated as follows:

$$Mw(AGU_{DA-CNF})\ (\frac{\mathrm{g}}{\mathrm{mol}}) = \frac{1}{\frac{\mathrm{DO}}{2} + \frac{\left(1 - \frac{DO}{2} \times Mw_{DA-AGU}\right)}{Mw_{AGU}}}\ (4)$$

Where DO (mol/g) is a degree of oxidation, which equals to amount of aldehyde groups in DA-CNF (mol/g), $\mathrm{Mw_{DA-AGU}}$ is Mw of oxidized AGU (160 g/mol), and $\mathrm{Mw_{AGU}}$ is Mw of AGU (162 g/mol).

An estimation of the degree of covalent functionalization will be explain using the example below (Bn-ON-CNF, prepared form DA-CNF wet cake with 4.8 mmol/g of aldehyde with 1.5 equiv. of Bn-$ONH_3^+Cl^-$, Supplementary Fig. S1). To perform an estimation, we calculated the intensity of the signal of the oxime carbon atoms (-C=N-O-R), which appears in the region 149-151 ppm and the total intensity of other signals corresponding to DA-CNF and R-ON-CNF 52-112 ppm (including oximated, oxidized and not oxidized glucose units). It is important to mention that a total intensity of each signal consists of the main peak and two side bands which appear 10 000 Hz apart on both sides of the main peak.

For the oxime carbon atom one of the side bands appear at ca. 227 ppm and has an intensity of 0.07 and the second overlaps with the signals form cellulose. The intensity of the second side band in not know, but is assumed to be equal to the intensity of the first side band. Thus, the total intensity of the oxime carbon atom ($I_{-C=N-O-Bn}$) equals to 1.14.

We then calculate the total intensity of signals corresponding to cellulose in the region of 52-112 ppm.. The total intensity of this region is 13.27. However, this region also includes side bands of the aromatic carbons and a signal from the benzylic carbon of $I_{Ph-CH2-ON}$ (ca. 70-75 ppm). To estimate the intensity of the signal corresponding to the benzylic carbon we calculated the total intensity of signals of all aromatic carbons, including the main band (116-143 ppm) and the side bands (200-224 ppm). The intensity of side bands was multiplied by 2 to account for the side bands overlapping with the signals of dialdehyde cellulose. The total intensity of the aromatic carbon atoms amounts to 5.69 (4.37+2*(0.16+0.5) = 5.69). The ratio between aromatic carbon atoms and benzylic carbon in benzylhydroxyl amine is 6/1, thus, the intensity of the benzylic carbon atom is estimated as 0.95 (5.69/6). The total intensity of the signals corresponding to cellulose in the region of 52-112 ppm (I_{CNF}) upon withdrawal of the intensity of the signals of the side bands and benzylic carbon and addition of the intensity of the signal corresponding to oxime carbon is estimated as 12.73 (13.27-0.95-(0.5+0.16+0.07)+1.14=12.73). We then can calculate molar ratio R_{mol} between the intensity of the signal corresponding to the oxime linkage and total intensity of the signals of AGU as follows:

$$R_{mol} = \frac{I_{-C=N-O-R}}{I_{DA-CNF}\ /N} = \frac{1.14}{12.8/6} = 0.537 \quad (5)$$

Where N (6) is number of carbon atoms in AGU of DA-CNF. The amount of oxime linkages installed per gram of DA-CNF can be calculated using average Mw of AGU from Table S2 as follows:

$$DF\ \left(\frac{\text{mmol}}{\text{g}}\right) = \frac{R_{mol}}{Mw_{AGU}} = \frac{0.537}{161.2} * 1000 = 3.3 \quad (6)$$

Integrated spectra of other R-ON-CNF film are presented in Appendix 2.

For Allyl-ON-CNF intensity of the signal of allylic carbon atom, which overlaps with the signals of other signals of CNF was calculated in the same fashion as for the benzylic carbon of Bn-ON-CNF, using signals of olefinic carbons instead of aromatic carbons.

For Me-ON-CNF the intensity of the signal of methyl carbon atom was taken equal to the intensity of the signal of the oxime carbon due to the absence of any other reference carbon atoms.

For pentyl-ON-CNF intensity of the signal of the carbon atoms overlapping with the signals of CNF were calculated using aliphatic signals in the region without the overlap (20-30 ppm).

For ArF_5-ON-CNF deconvolution of the signal in the region 136-160 ppm was performed in order to calculate the portion of the intensity of the peak corresponding to oxime carbon signal at 150.2 ppm (0.463).

It is important to note that all the calculations were performed assuming that substrate consists of cellulose, however hemicellulose is also present in the substrate. Due to the difficulties in estimation of the portion of hemicellulose, calculations were performed as described.

Measurements of water uptake of DA-CNF and R-ON-CNF

A piece of dried film (30-60 mg) was weighted ($m_{\mathrm{CNF\ film\ dry}}$) and placed in a vial filled with water. The film was left in water for 24 hours. The film was removed from the water and wiped with a filter paper to remove water from the surface of the film. The film was weighted again ($m_{\mathrm{CNF\ film\ wet}}$). Water uptake was calculated as follows:

$$Water\ uptake, \% = \frac{m_{CNF\ film\ wet}}{m_{CNF\ film\ dry}} \quad (7)$$

Defunctionalization of R-ON-CNF film

For kinetic studies a piece of CF_3-Bn-ON-CNF film (0.1345g) was placed into solution, containing 0.0945 g of pentafluorobenzyl bromide as an internal standard (IS), 5.8 g acetone, 5.8 g D_2O and HCl (1M). To estimate the amount of CF_3-Bn- moiety released into the solution an aliquot of the solution was taken after different periods of time and subjected to ^{19}F NMR. An amount of the released fragment was calculated using signal corresponding to CF_3 group (-63.0 ppm) in relation to one of the signals of IS (-155.2), for a representative ^{19}F NMR spectra (after 11 hours) see Appendix 3.

To analyze the structure of the detached moiety, upon the completion of the reaction the reaction mixture was extracted with DCM/water and the organic phase was analyzed by ^{1}H MNR after the evaporation of the solvent (Supplementary Fig. S2).

The analysis of the reaction mixture revealed a presence of two major species of the detached fragment: O-(p-trifluoromethylbezyl)hydroxylamine hydrochloride (indicated by signal of benzylic protons at δ 5.10 ppm) and the product of it reaction with acetone (indicated by signal of benzylic protons at δ 4.77 ppm and signals of methyl groups at 1.87 and 1.91 ppm).

Defunctionalization of Bn-ON-CNF-2h film was performed in acetone/water (1/1, v/v) HCl (1.2M) solutions for 36 hours at room temperature. The recovered films were washed with acetone and water, dried at 93 °C under reduced pressure and subjected to tensile testing. To assure a complete detachment of the benzyl group after the tensile testing the films were cut into small pieces (ca. 1mm*1mm) and subjected to solid-state NMR measurements (see Appendix 2, Defunctionalized-Bn-ON-CNF).

Triboelectric measurements

The TENGs were fabricated in a three steps process, 1) application of conductive silver paste on the required area (2.5 x 1.3 cm^2) of CNF films (thickness ~100-150 µm) and drying in the oven at 80 °C for 2h, 2) attaching copper tape electrodes on the conductive area, and 3) soldering a copper wire to the copper electrodes for output measurement. The vertical contact separation mode TENGs were then realized by mounting two different parts of TENG on a linear motor setup, where one part is fixed on a linear motor stopper and the second part is attached to the linear motor end. These two parts are brought into contact by a periodic motion of linear motor from a distance of 2 cm with a frequency of ~1.8 Hz. The output voltage is measured using Keithley's DMM 7510.

Supplementary Figures

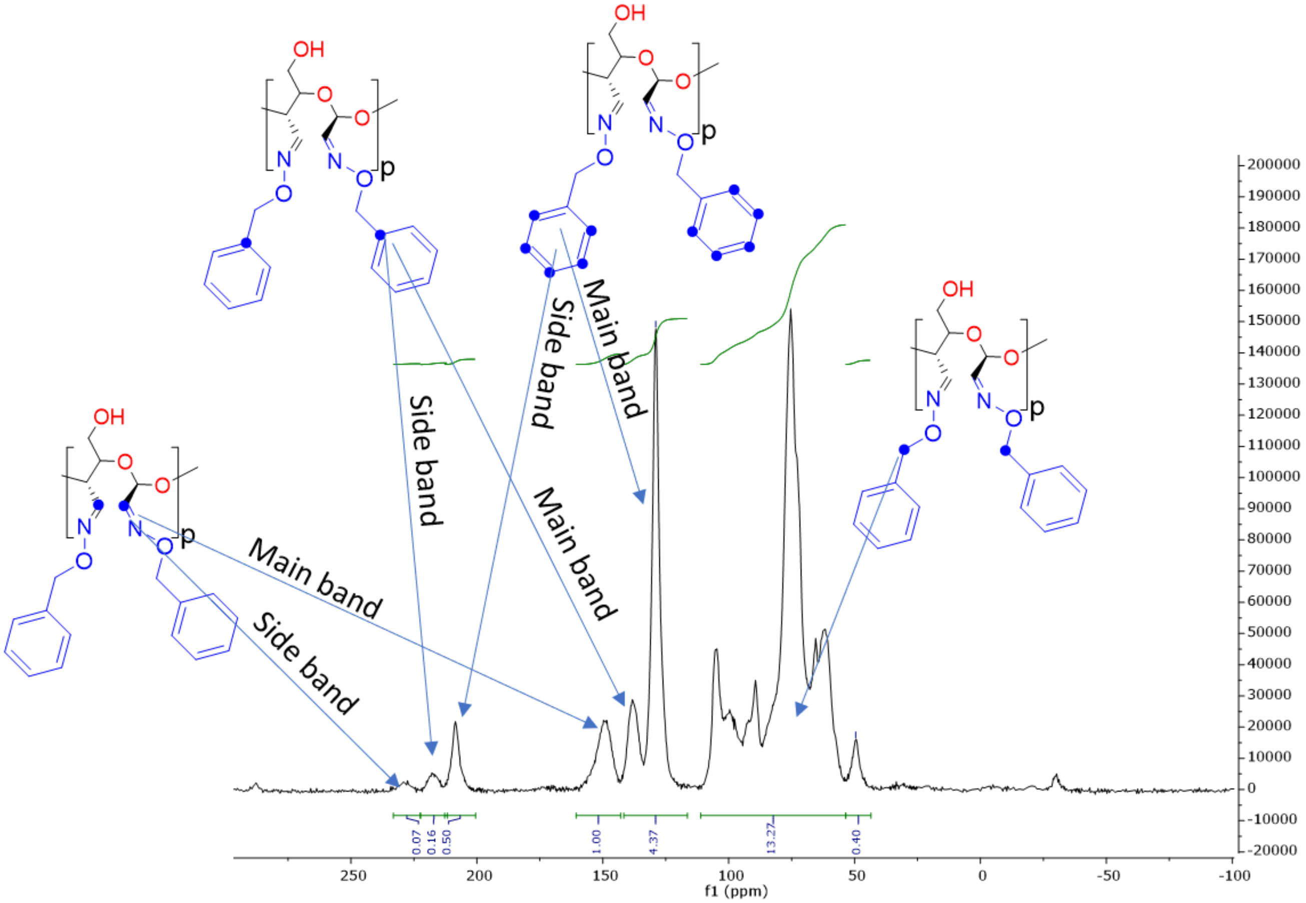

Supplementary Fig. 1. Signal assignment in ^{13}C CP/ MAS NMR of Bn-ON-CNF film.

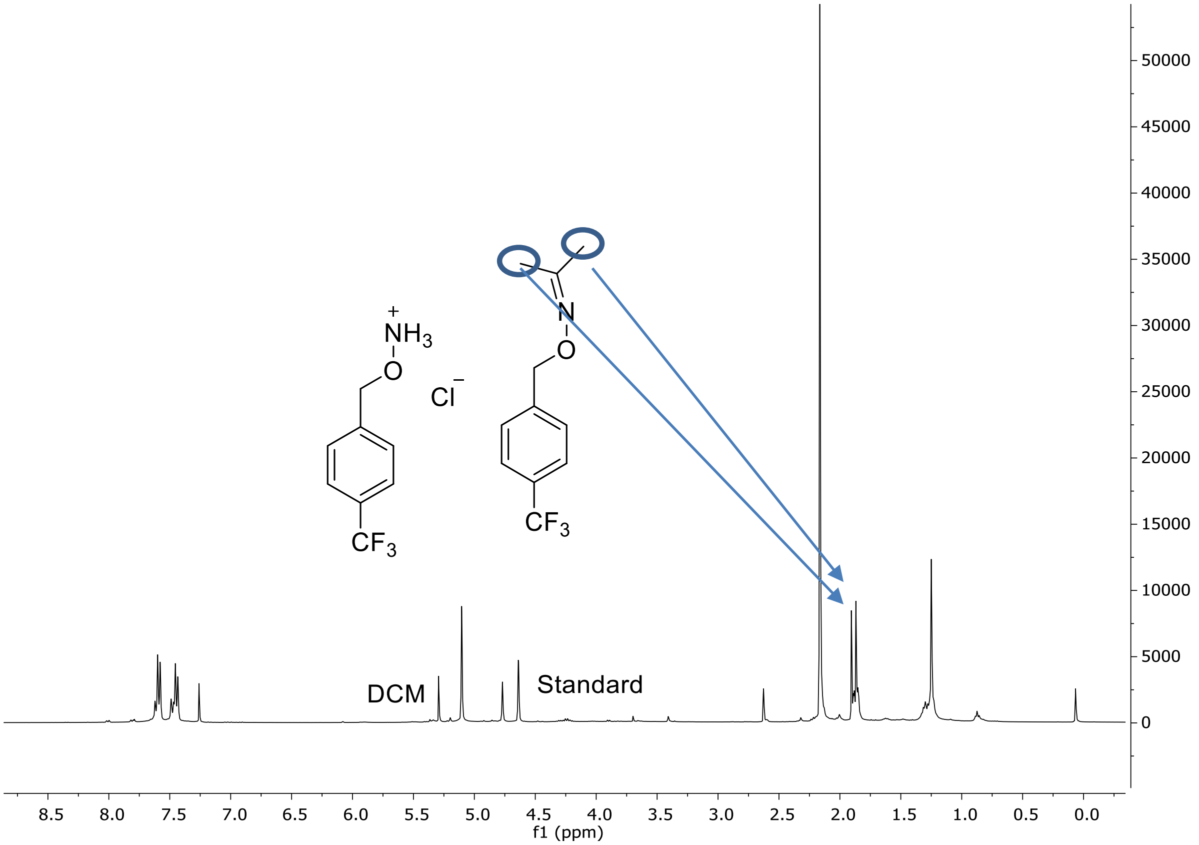

Supplementary Fig. 2. ^{1}H NMR spectrum in $CDCl_3$of the organic phase recovered upon DCM/water extraction of the mixture obtained during the detachment experiment with CF_3Bn-ON-CNF film.

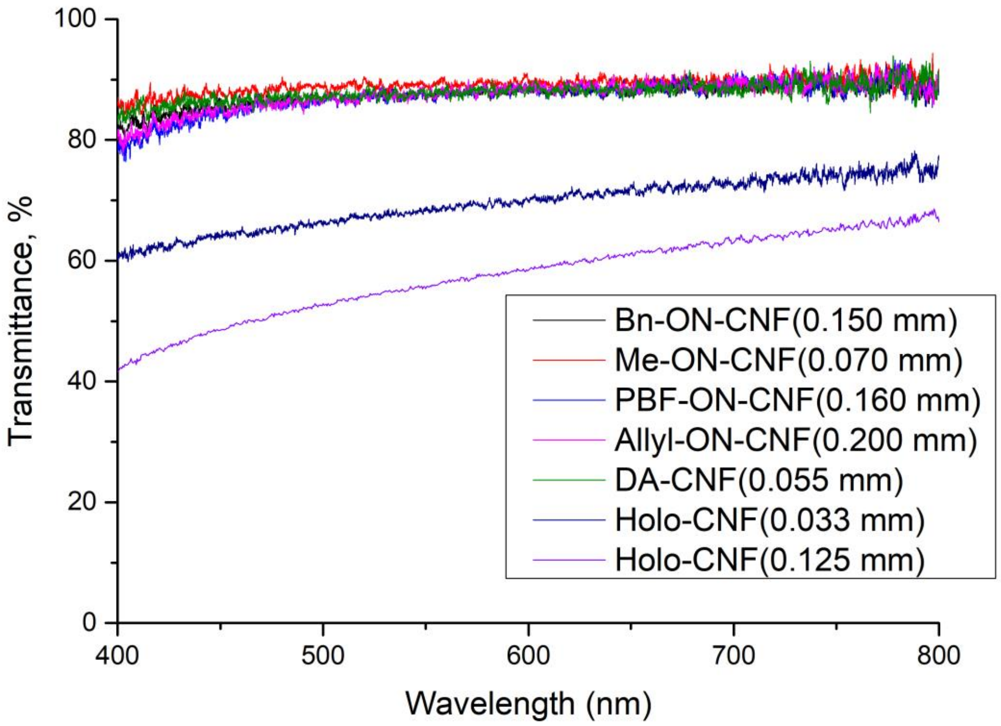

Supplementary Fig. 3. Optical transmittance of Holo-CNF, DA-CNF, and selected R-ON-CNF films in visible light range. Source data are provided as a Source Data file.

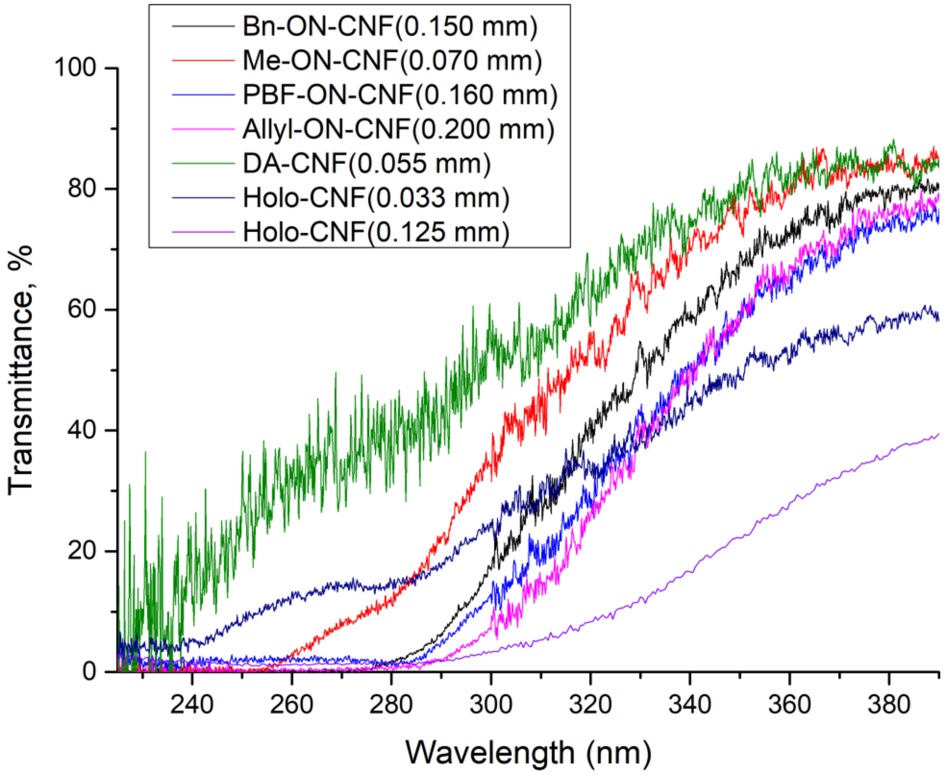

Supplementary Fig. 4. Optical transmittance of Holo-CNF, DA-CNF, and selected R-ON-CNF films in UV range. Source data are provided as a Source Data file.

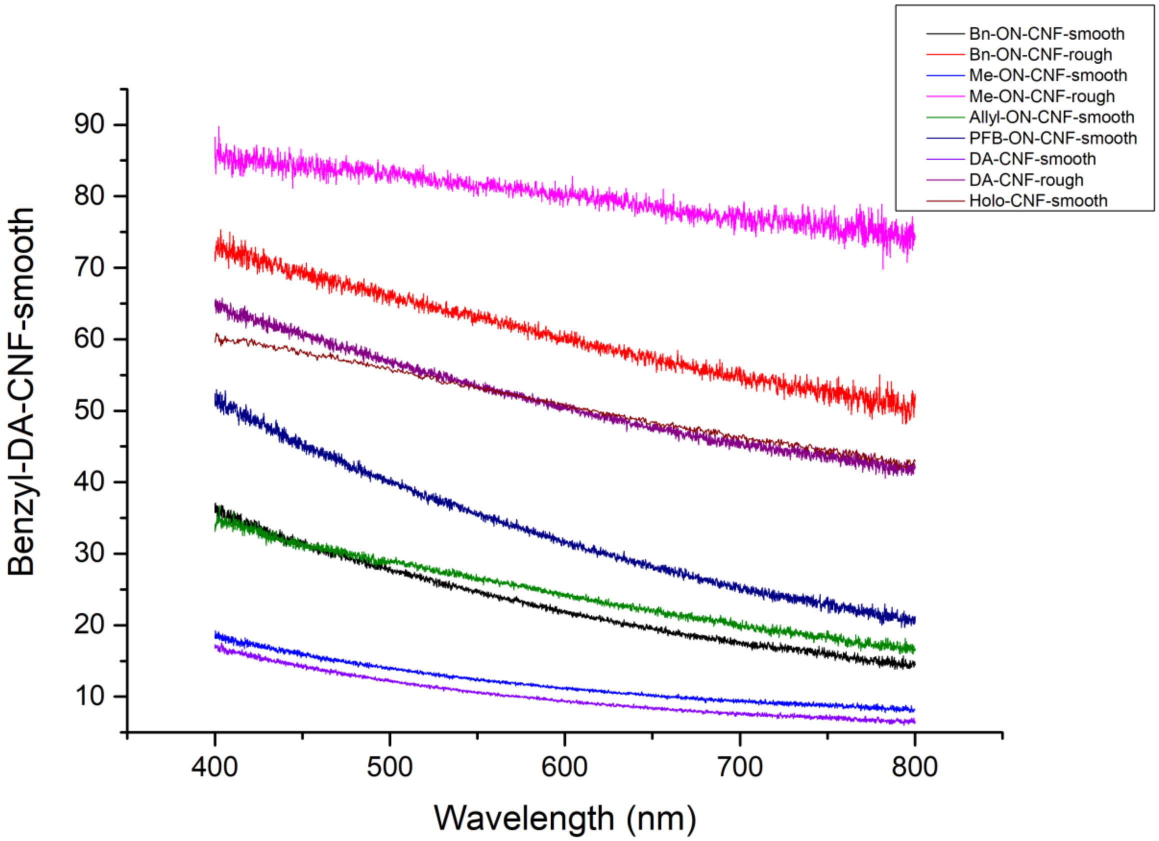

Supplementary Fig. 5. Haze of Holo-CNF, DA-CNF, and selected R-ON-CNF films prepared using either smooth or rough surface. Source data are provided as a Source Data file.

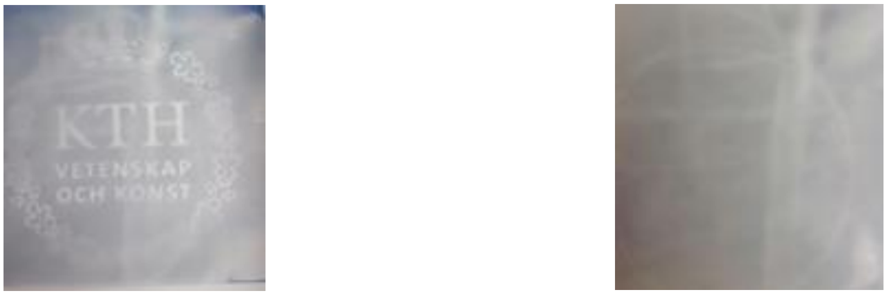

a.

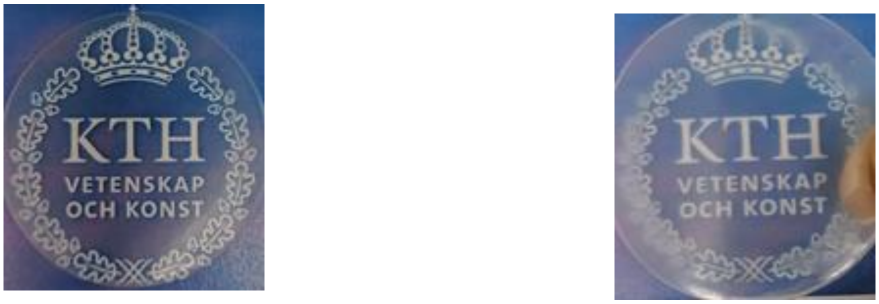

b.

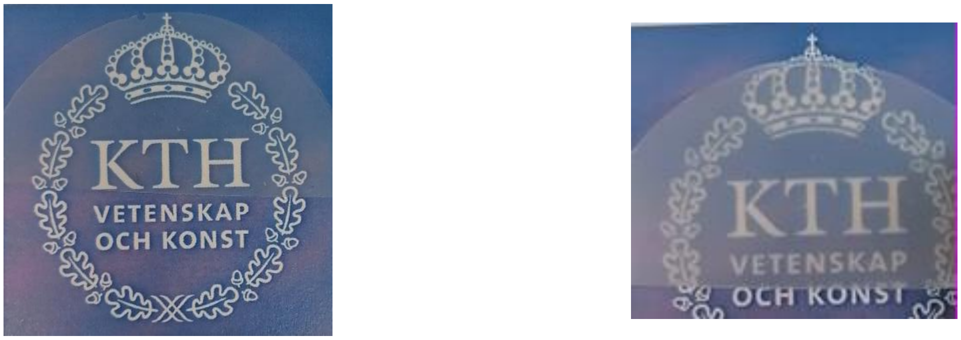

c.

Supplementary Fig. 6. Pristine and modified CNF films on the surface (left) and lifted a few cm from the surface (right) for a. Holo-CNF, b. DA-CNF, c. Bn-ON-CNF.

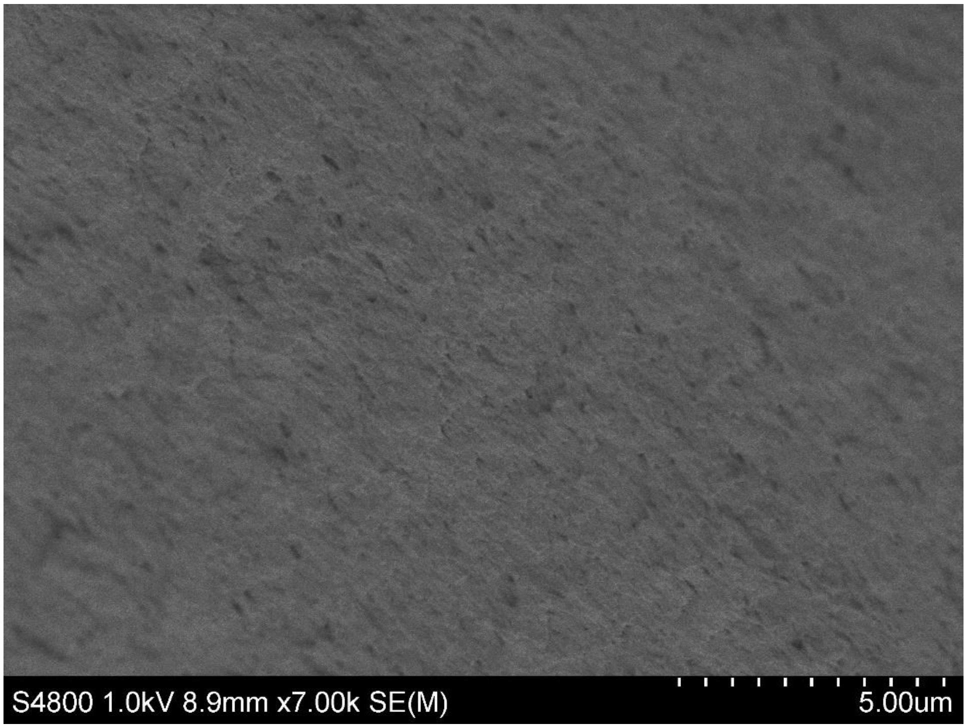

Supplementary Fig. 7. SEM image of Bn-ON-CNF film (cross section).

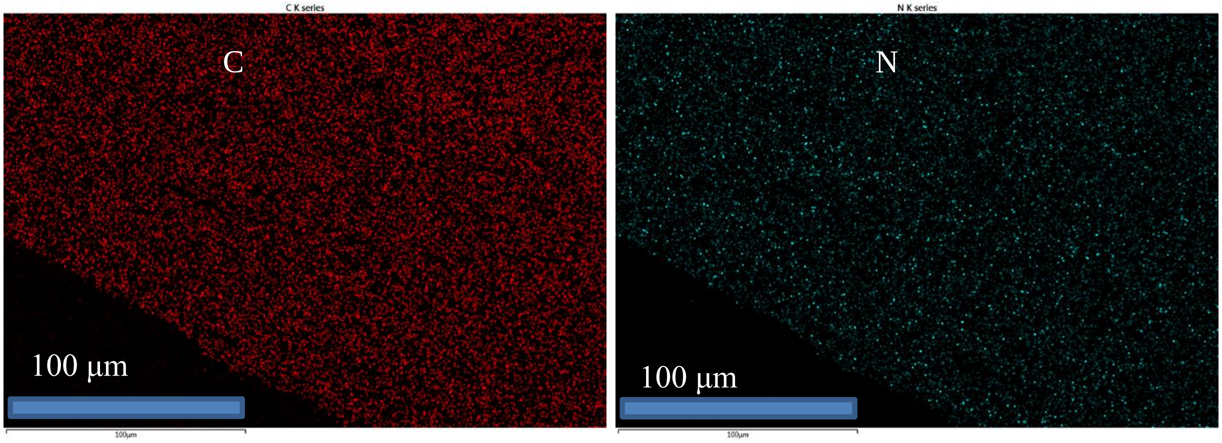

Supplementary Fig. 8. SEM/EDS images of Bn-ON-CNF films (cross section) showing distribution of carbon (left) and nitrogen (right).

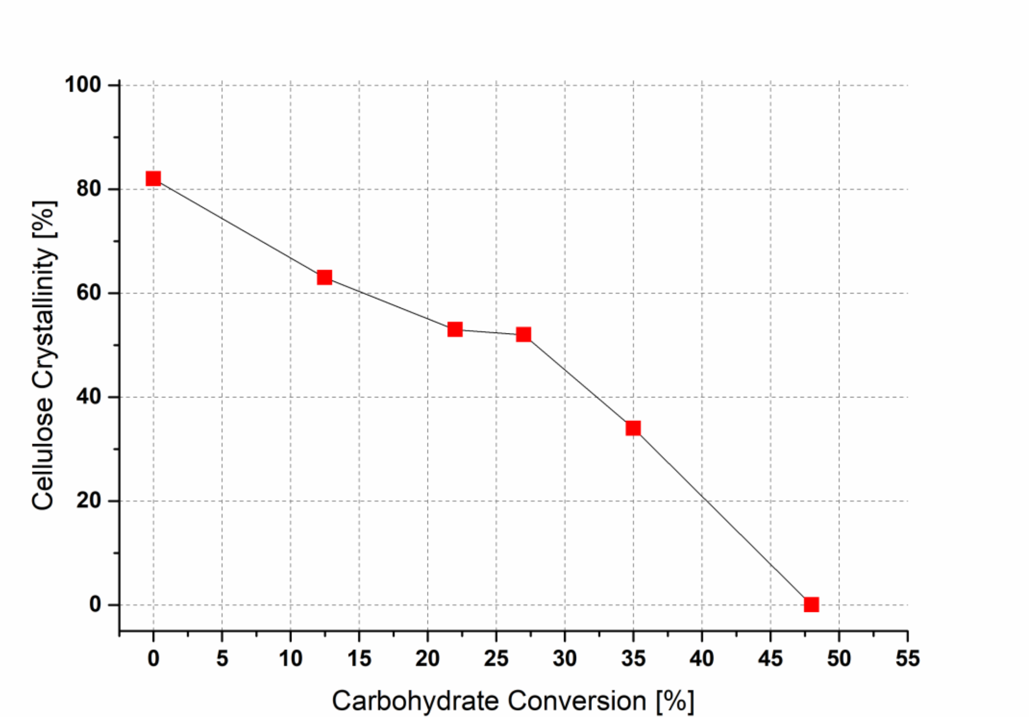

Supplementary Fig. 9. Decrease in crystallinity of cellulose as a function of oxidation conversion, showing two distinct regimes of decrease.

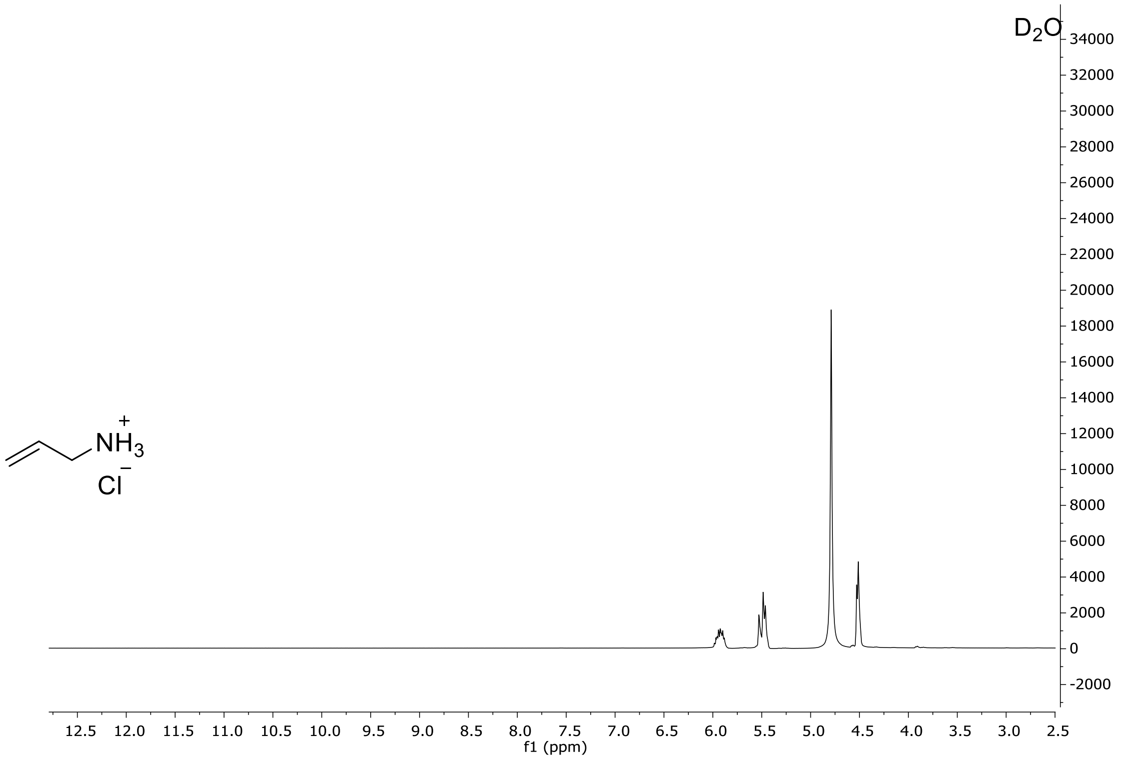

Supplementary Fig. 10. ^{1}H NMR spectrum of the starting material.

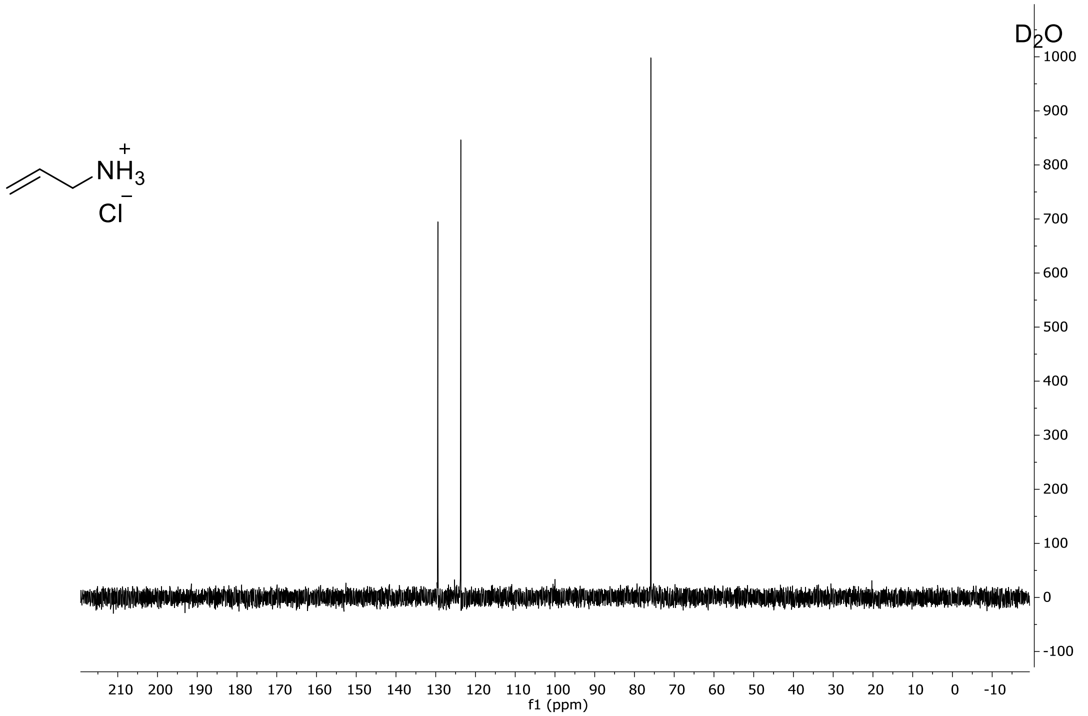

Supplementary Fig. 11. ^{13}C NMR spectrum of the starting material.

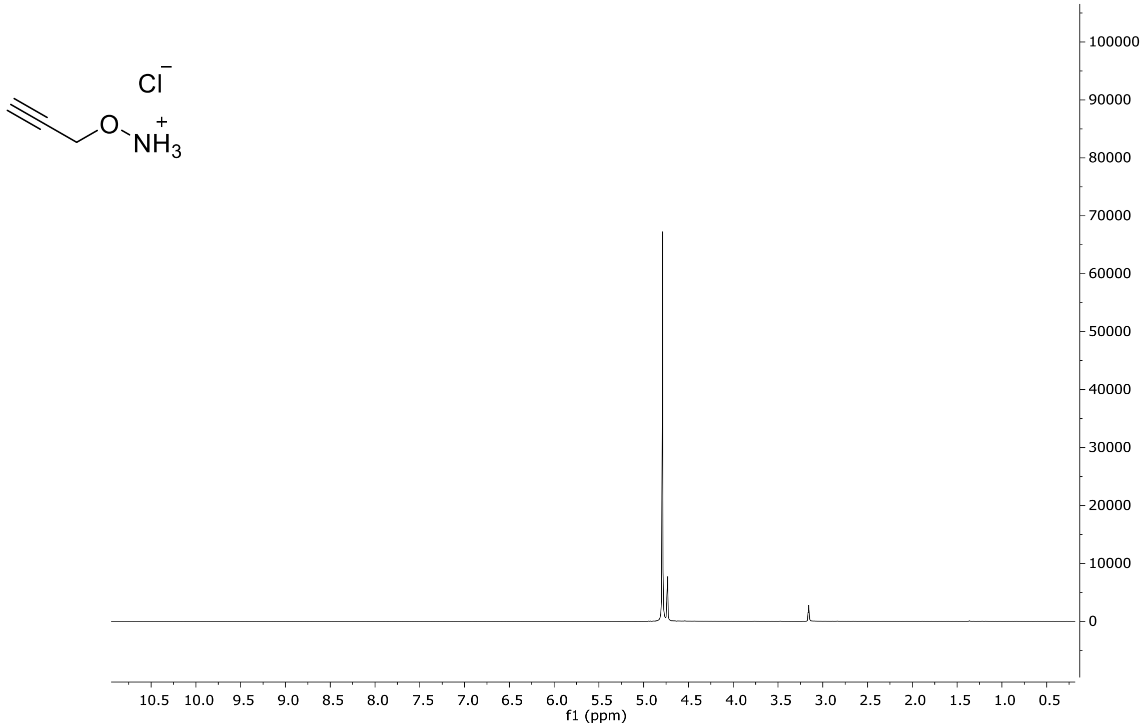

Supplementary Fig.12. ^{1}H NMR spectrum of the starting material.

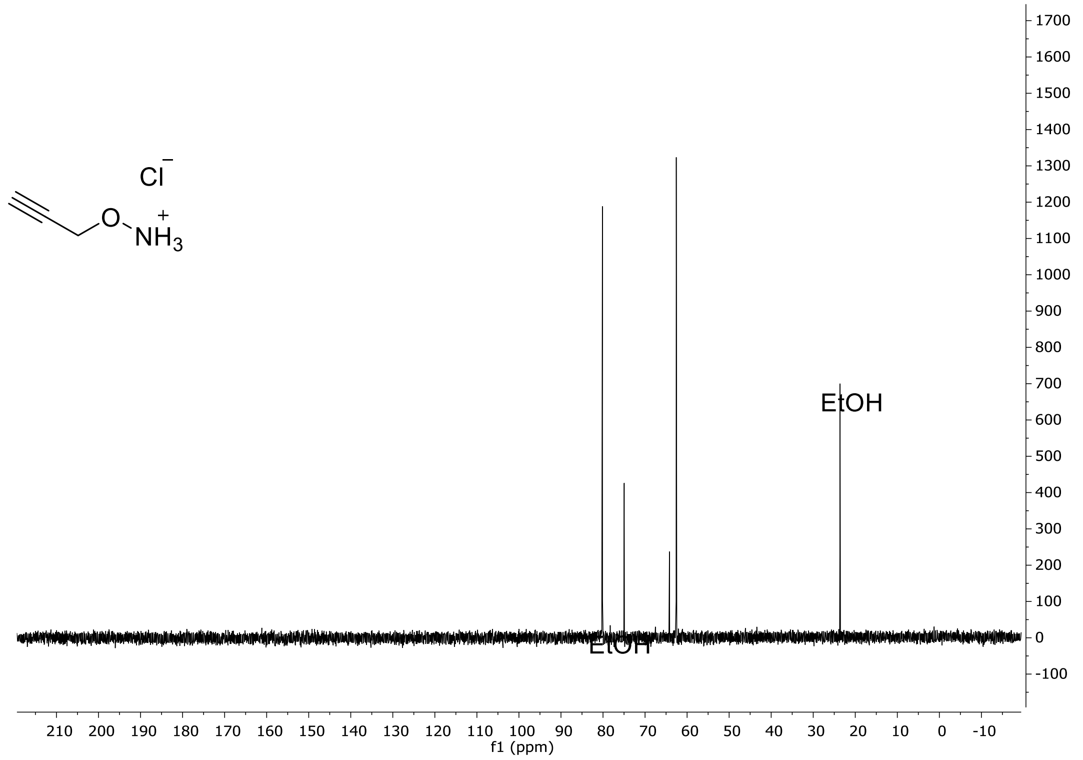

Supplementary Fig. 13. ^{13}C NMR spectrum of the starting material.

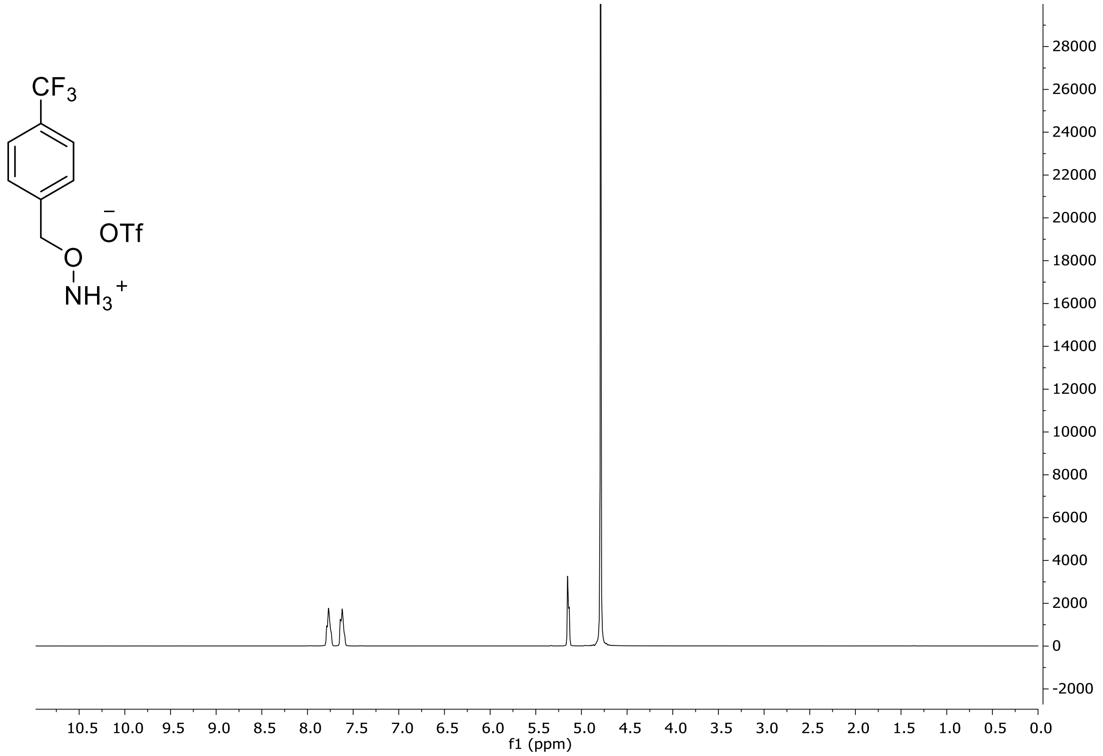

Supplementary Fig. 14. ^{1}H NMR spectrum of the starting material.

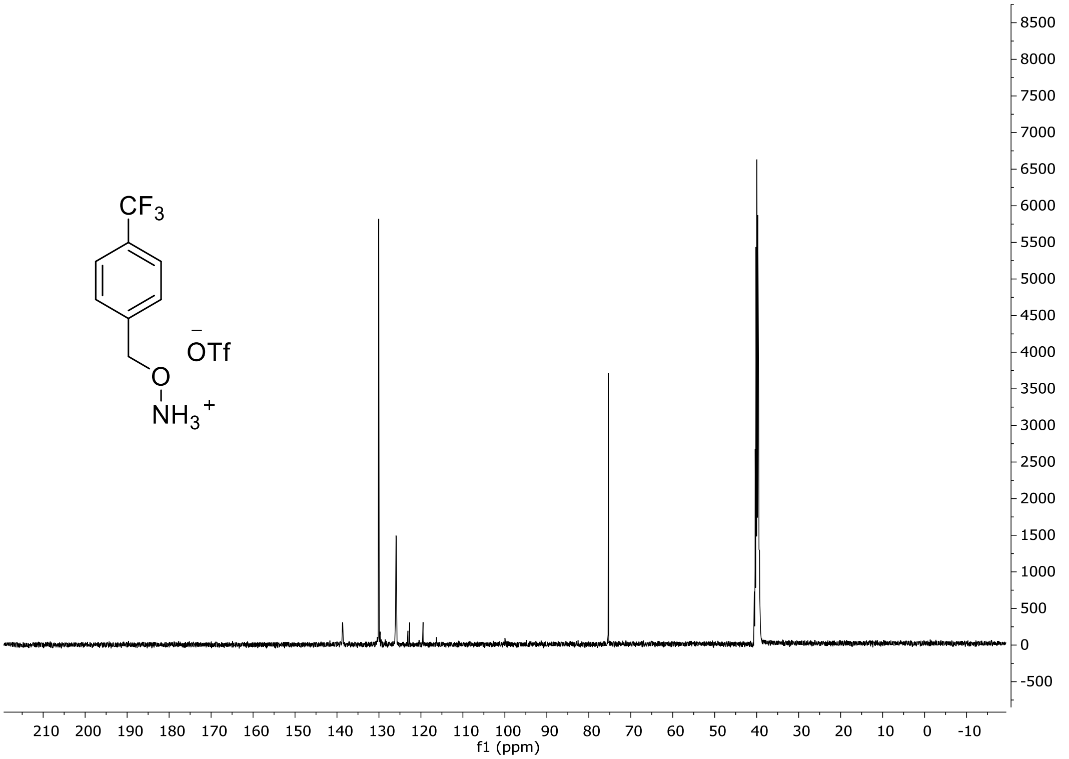

Supplementary Fig. 15. ^{13}C NMR spectrum of the starting material.

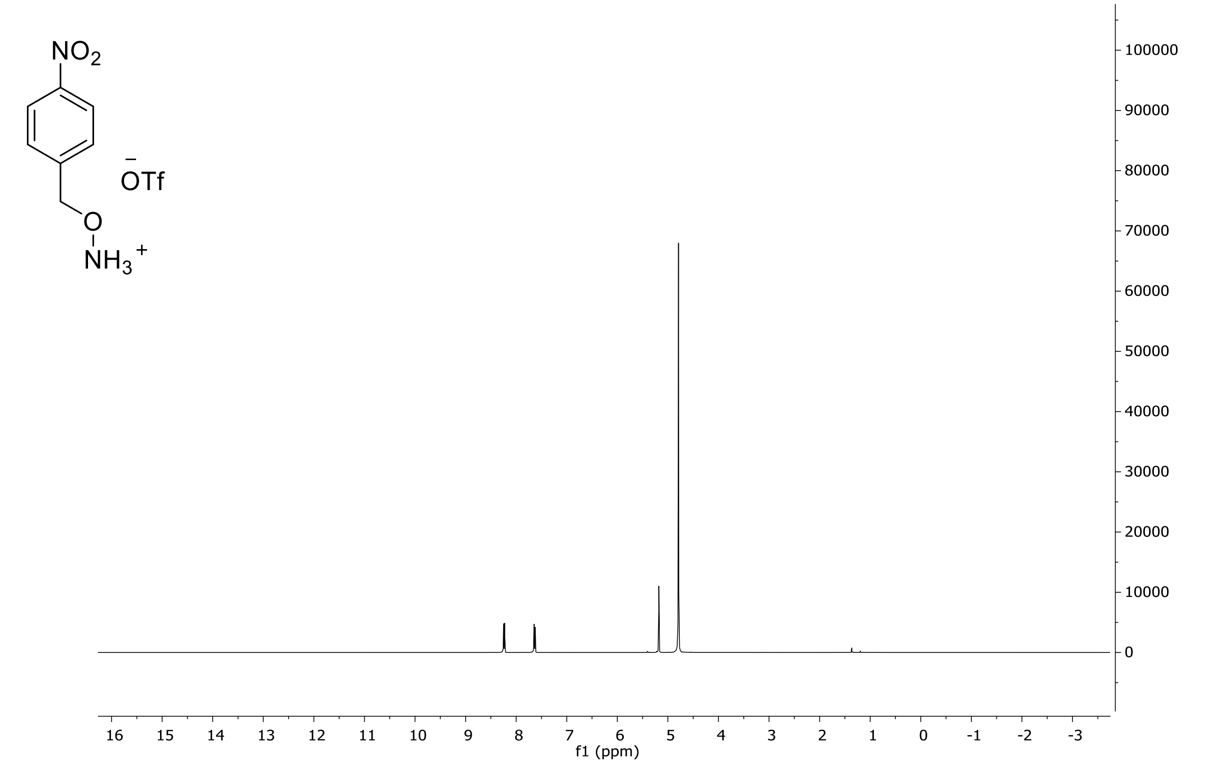

Supplementary Fig. 16. ^{1}H NMR spectrum of the starting material.

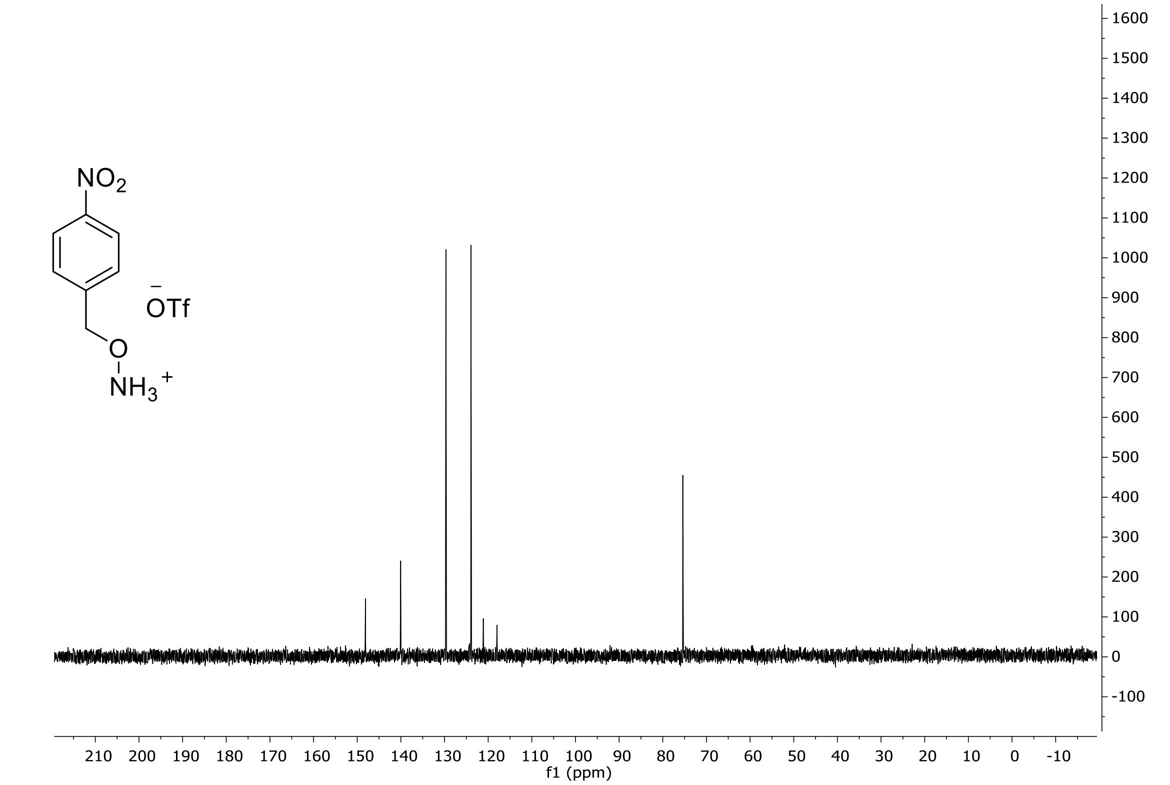

Supplementary Fig. 17. ^{13}C NMR spectrum of the starting material.

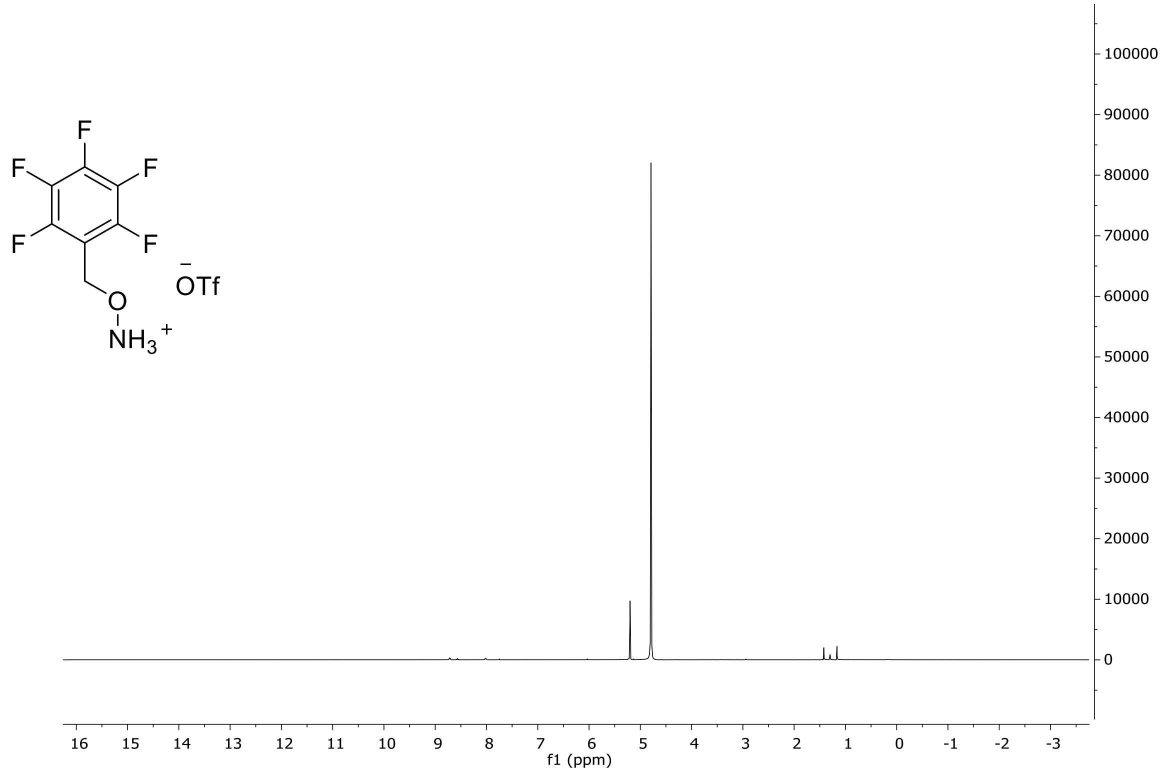

Supplementary Fig. 18. ^{1}H NMR spectrum of the starting material.

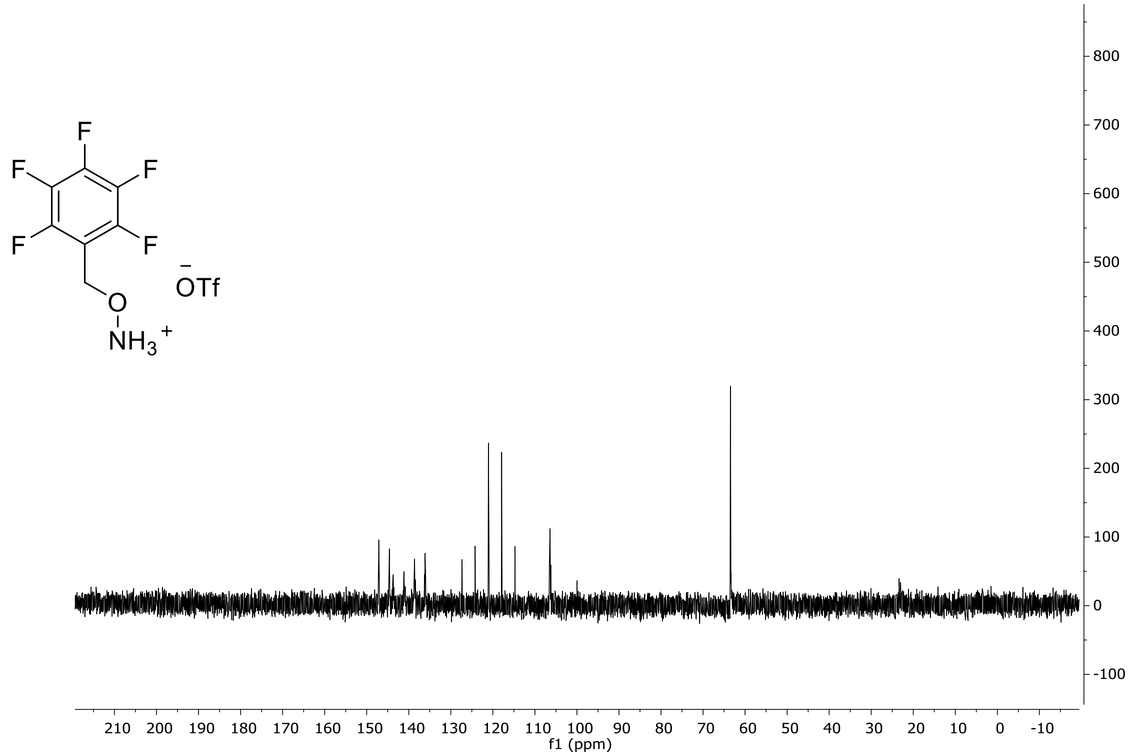

Supplementary Fig. 19. ^{13}C NMR spectrum of the starting material.

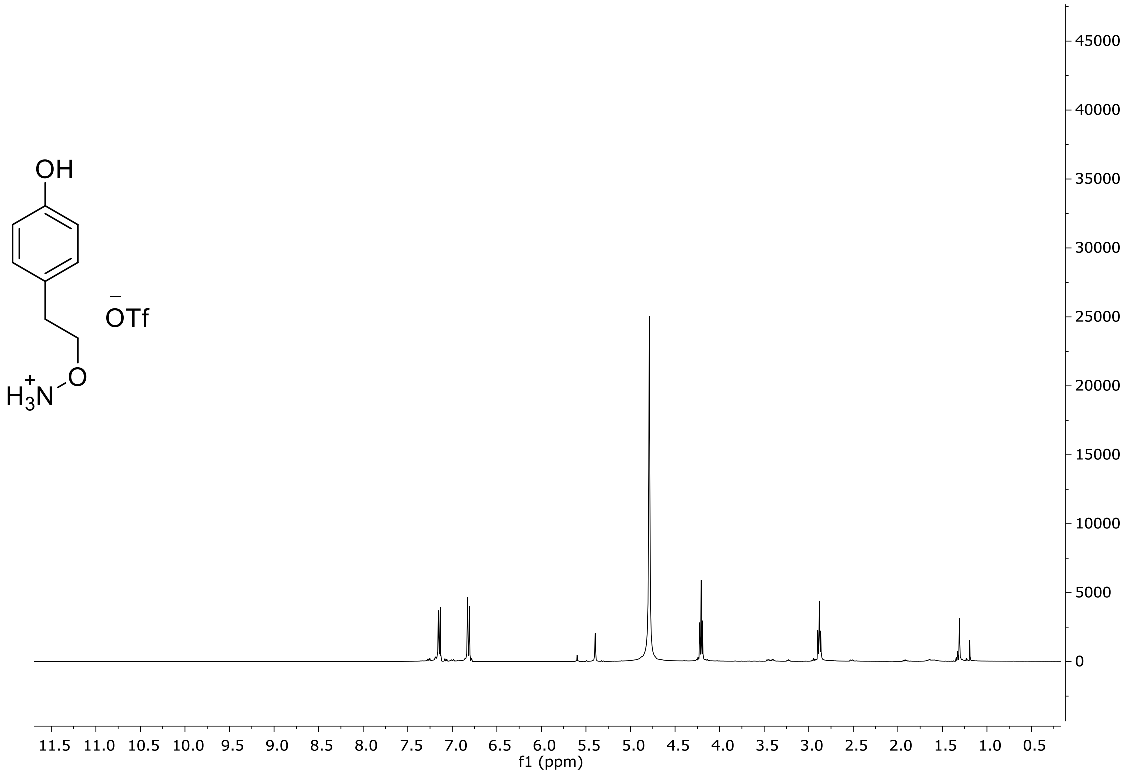

Supplementary Fig. 20. ^{1}H NMR spectrum of the starting material.

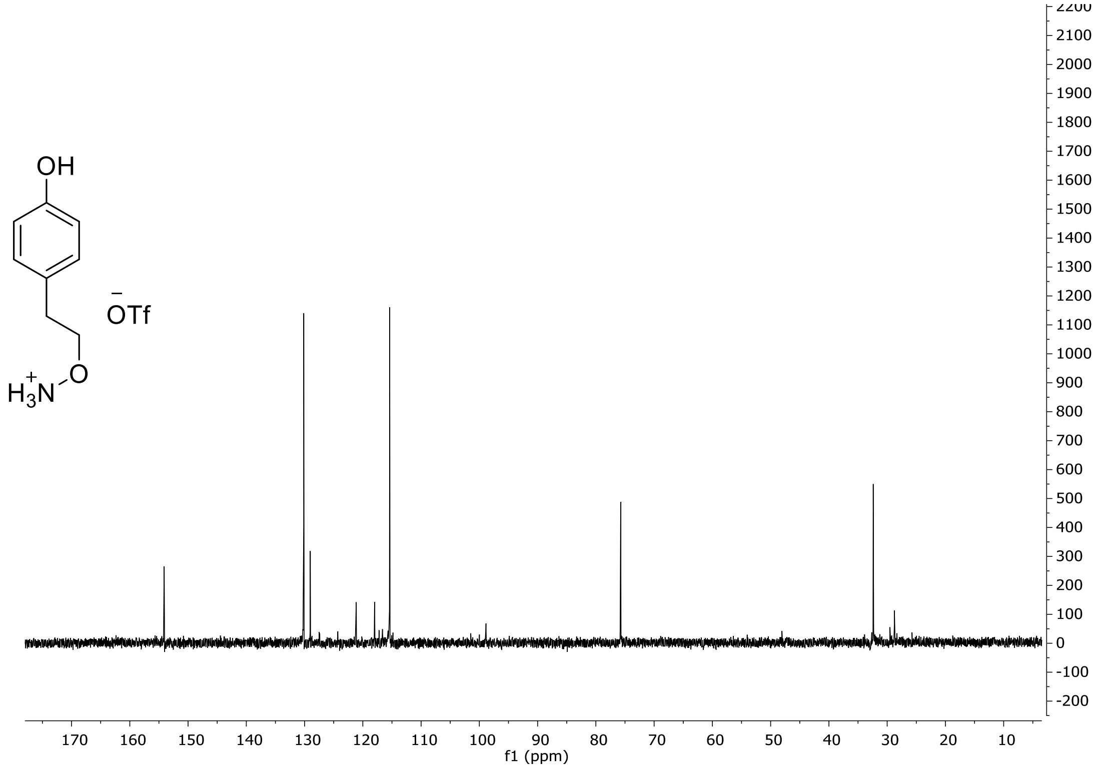

Supplementary Fig. 21. ^{13}C NMR spectrum of the starting material.

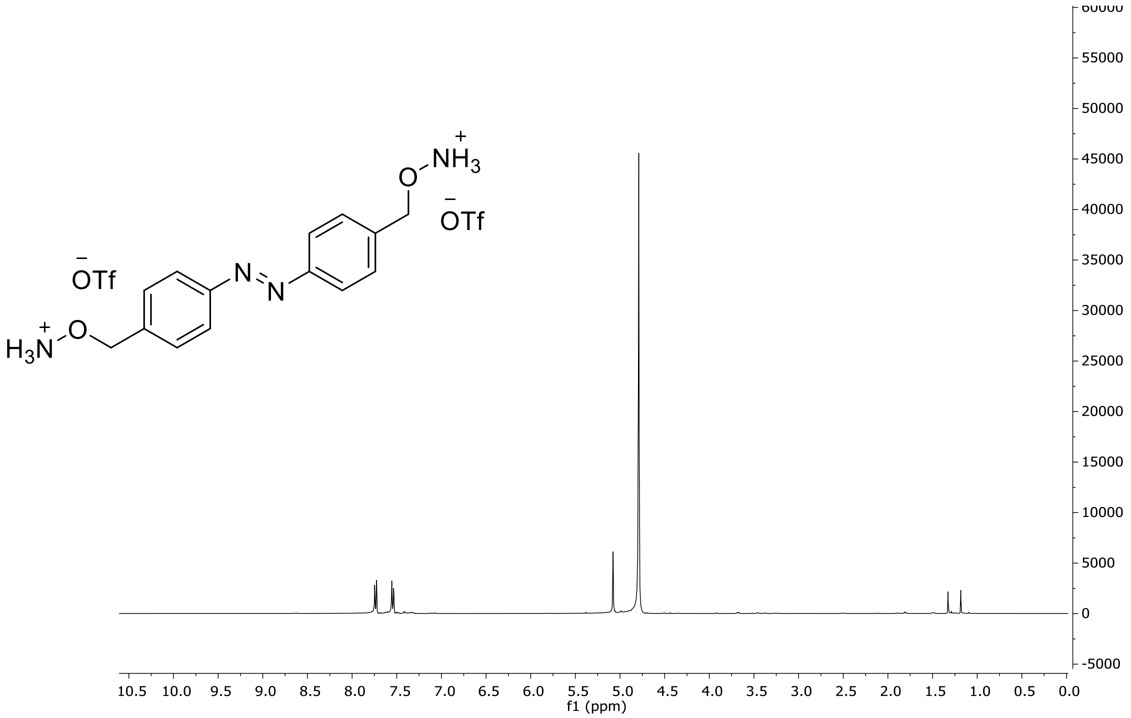

Supplementary Fig. 22. ^{1}H NMR spectrum of the starting material.

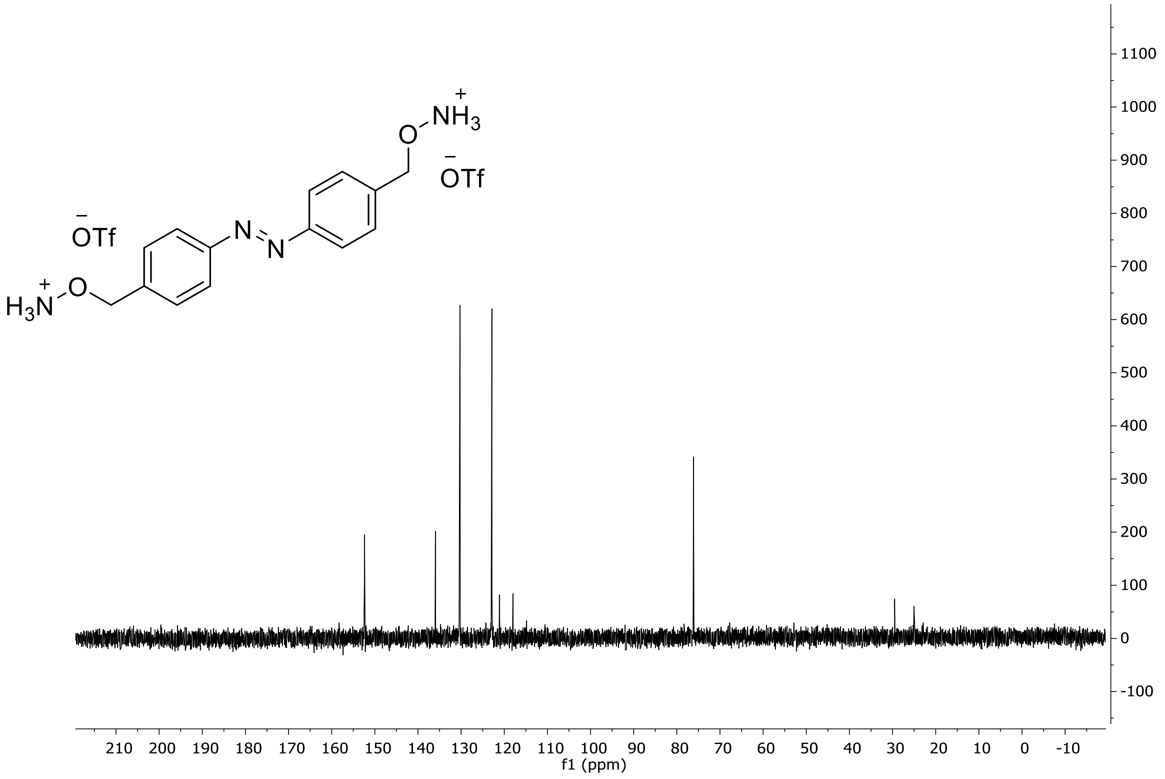

Supplementary Fig. 23. ^{13}C NMR spectrum of the starting material.

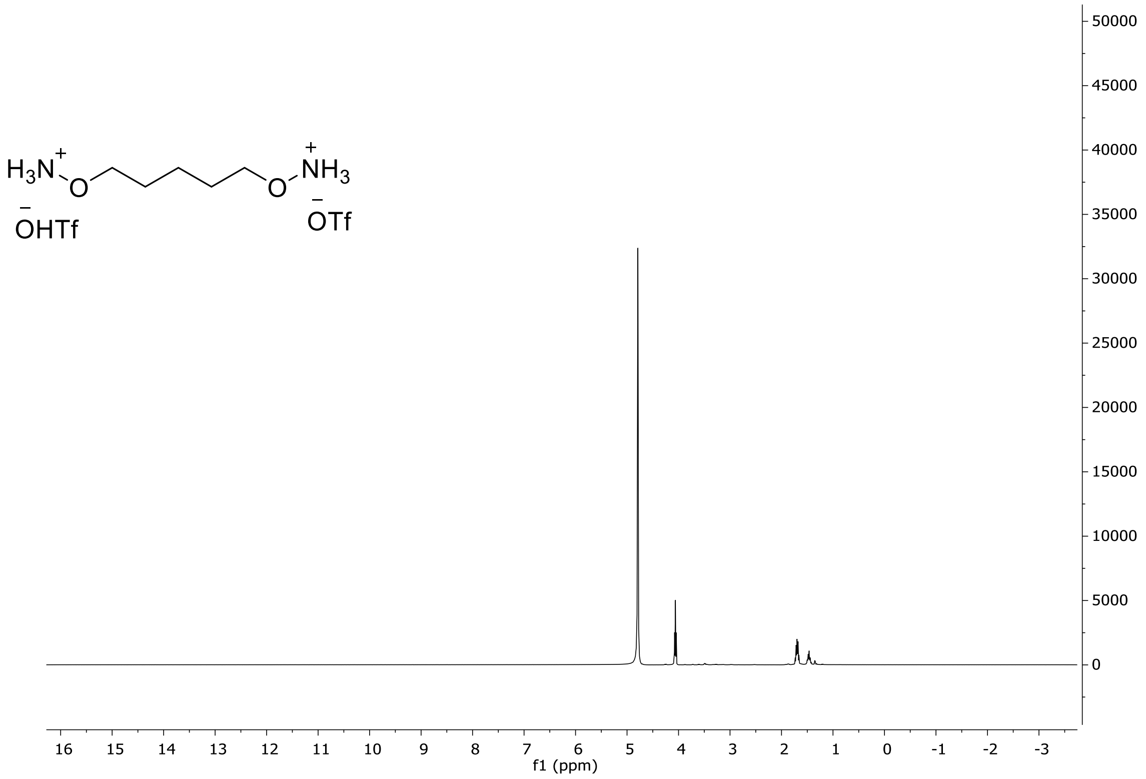

Supplementary Fig. 24. ^{1}H NMR spectrum of the starting material.

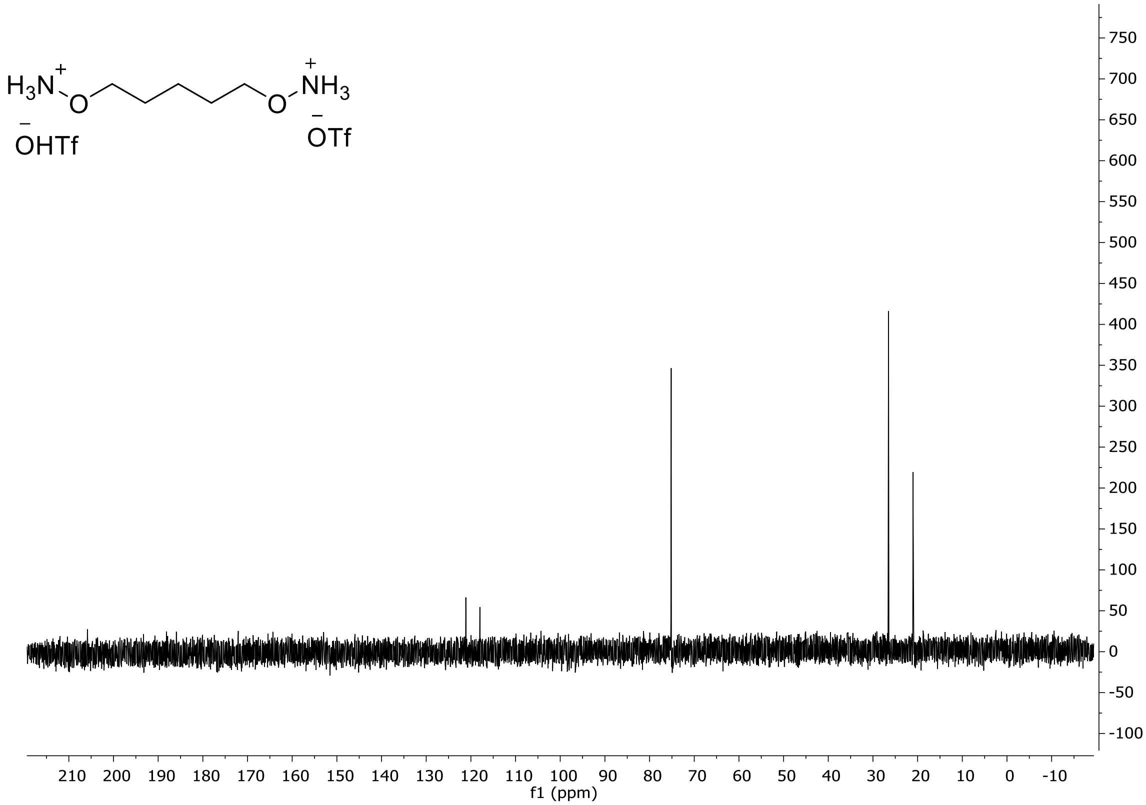

Supplementary Fig. 25. ^{13}C NMR spectrum of the starting material.

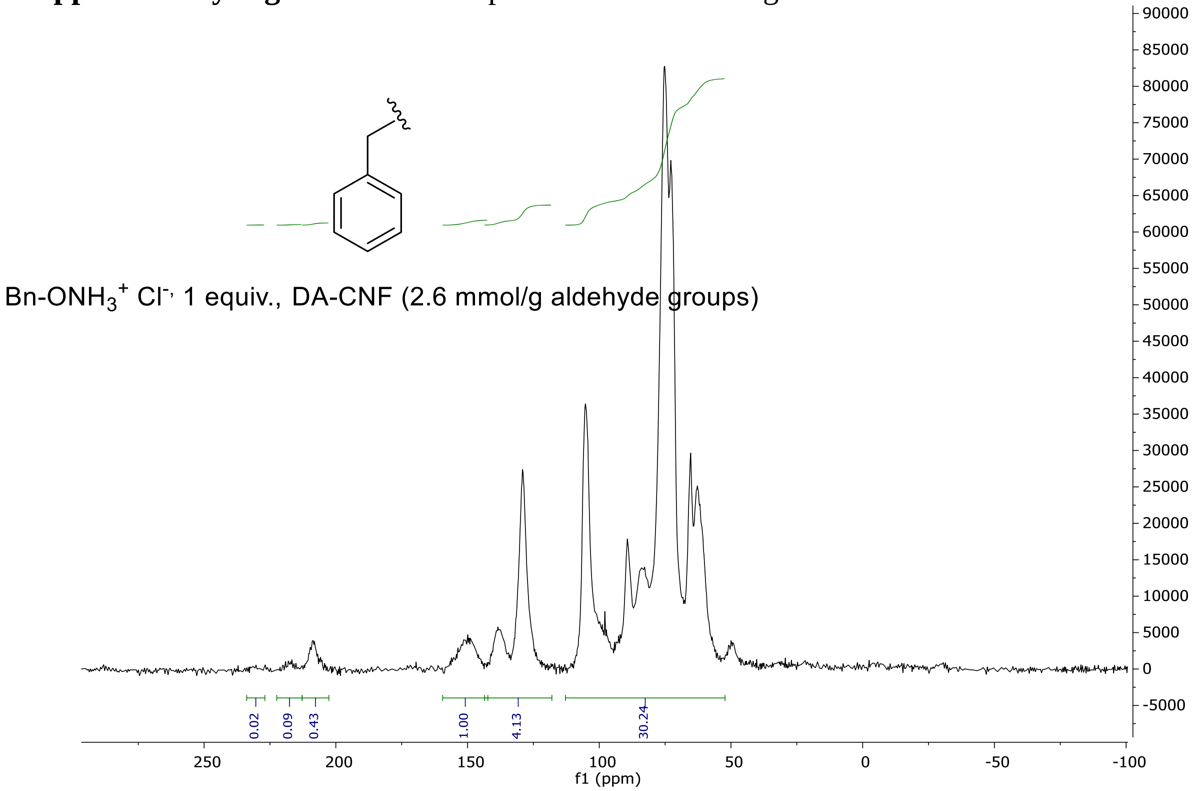

Supplementary Fig. 26. CP/MAS ^{13}C NMR spectrum of Bn-ON-CNF.

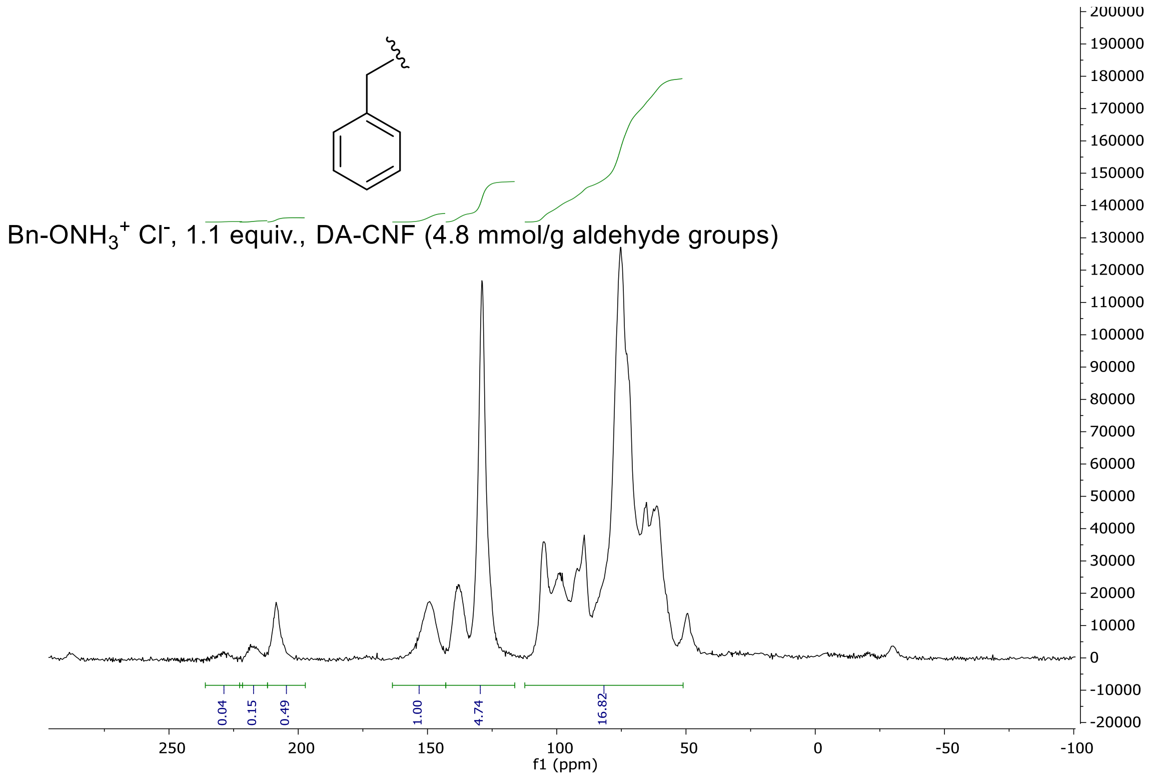

Supplementary Fig. 27. CP/MAS ^{13}C NMR spectra of Bn-ON-CNF.

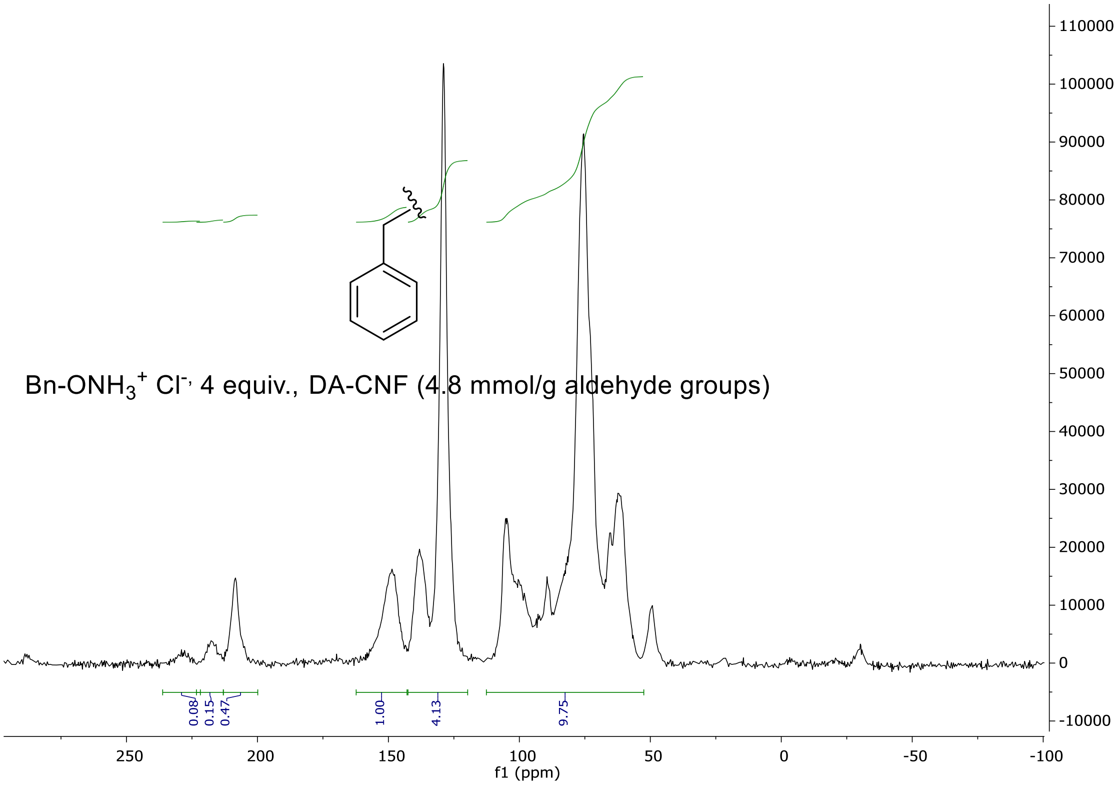

Supplementary Fig. 28. CP/MAS ^{13}C NMR spectra of Bn-ON-CNF.

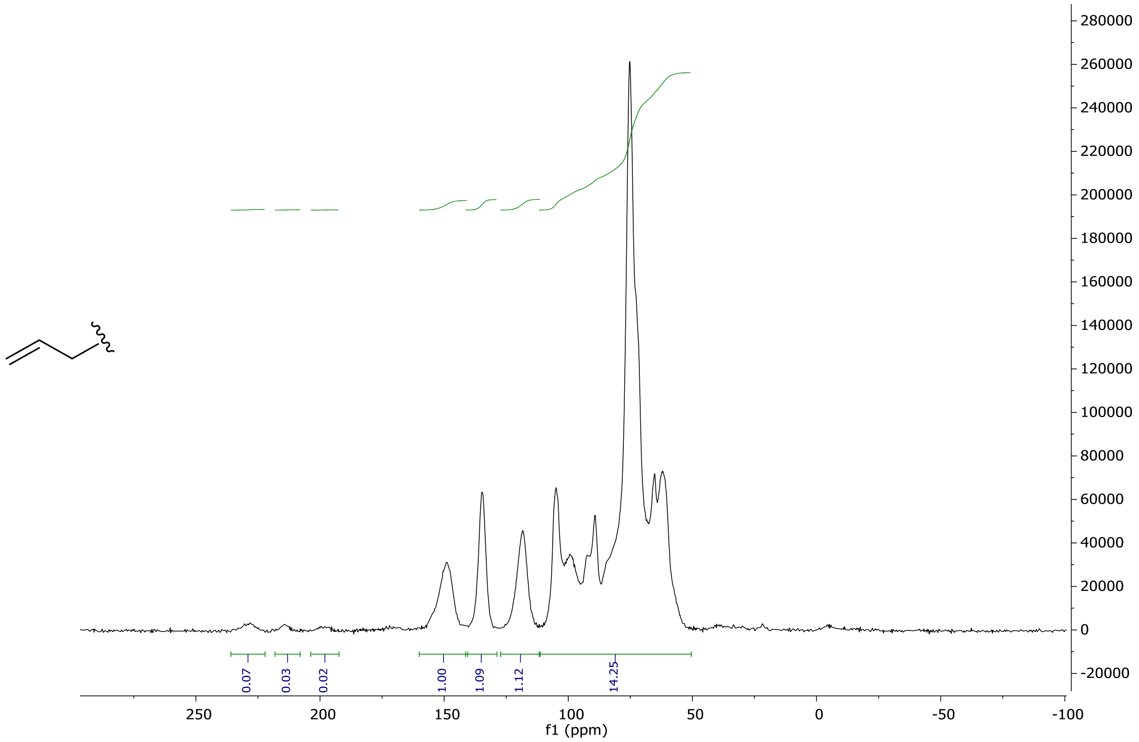

Supplementary Fig. 29. CP/MAS ^{13}C NMR spectra of Allyl-ON-CNF.

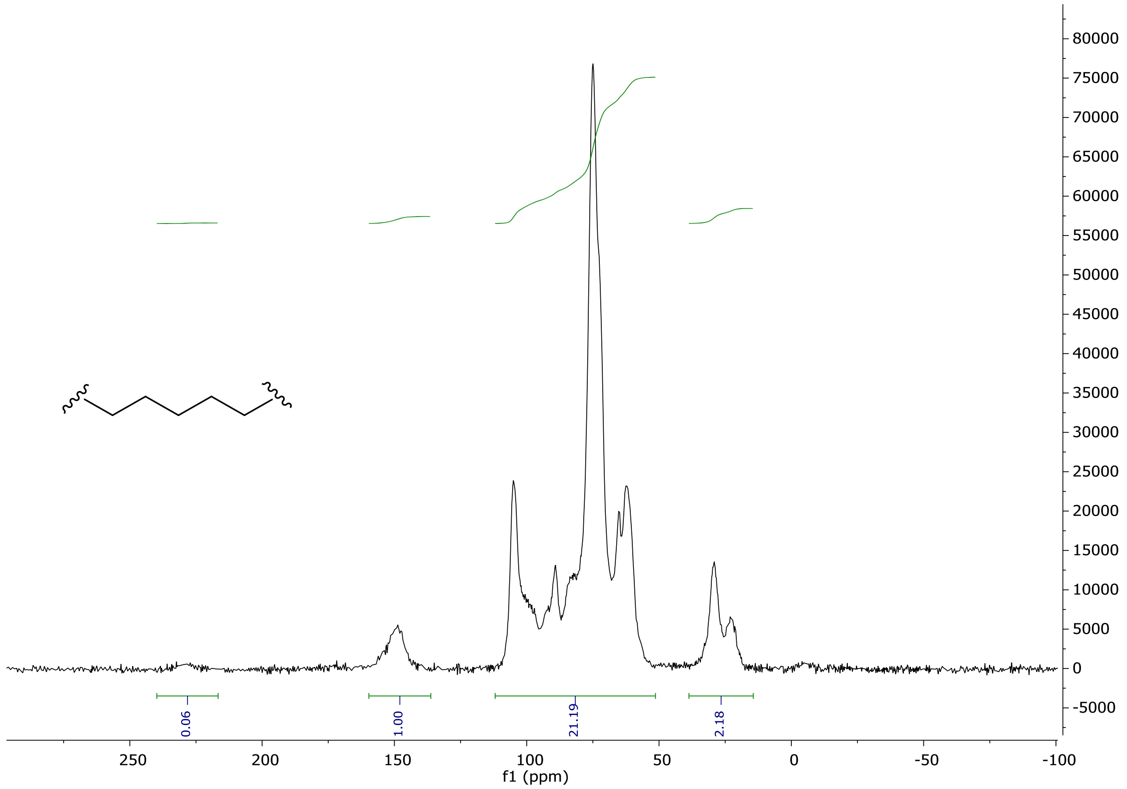

Supplementary Fig. 30. CP/MAS ^{13}C NMR spectra of pentyl-ON-CNF.

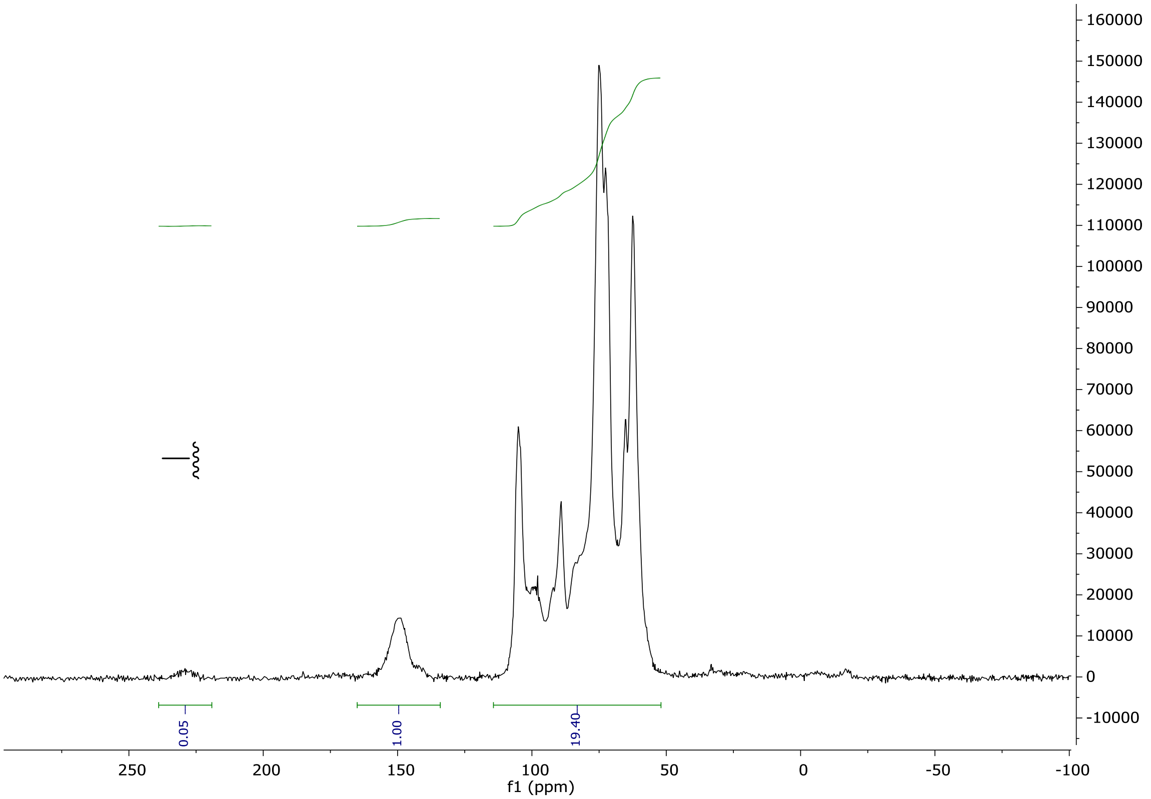

Supplementary Fig. 31. CP/MAS ^{13}C NMR spectra of Me-ON-CNF.

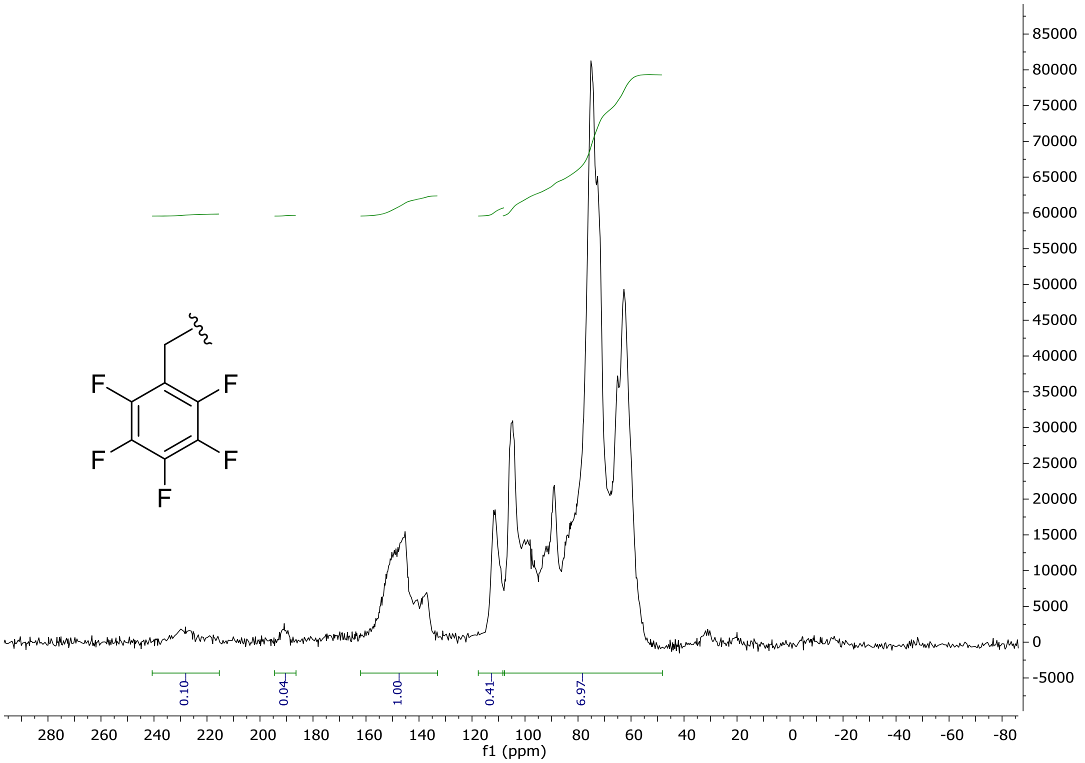

Supplementary Fig. 32. CP/MAS ^{13}C NMR spectra of PFB-ON-CNF.

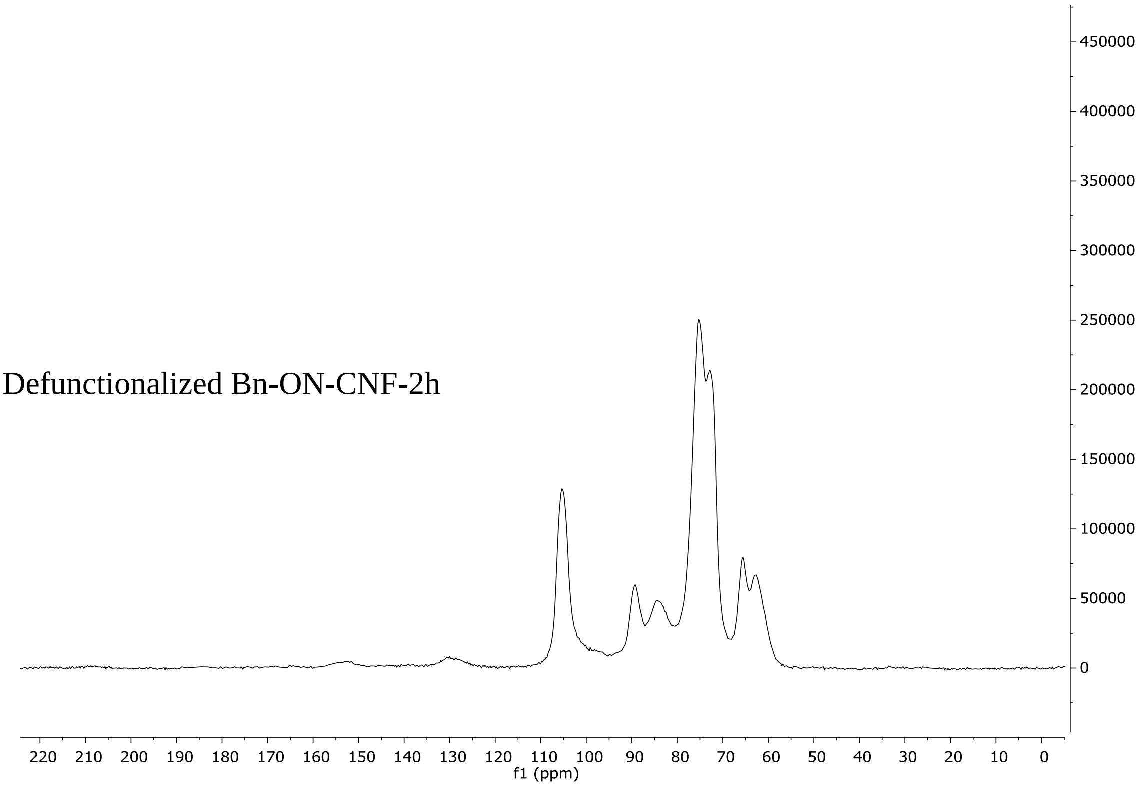

Supplementary Fig. 33. CP/MAS ^{13}C NMR spectra of defunctionalized Bn-ON-CNF.

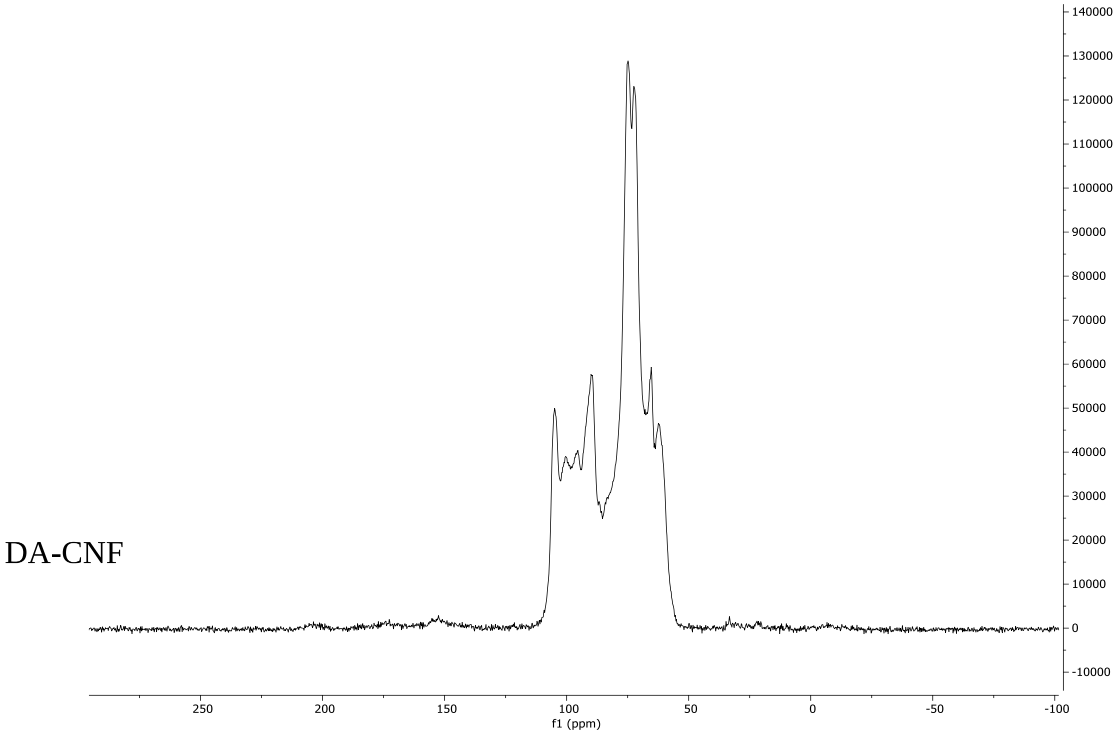

Supplementary Fig. 34. CP/MAS ^{13}C NMR spectra of DA-CNF.

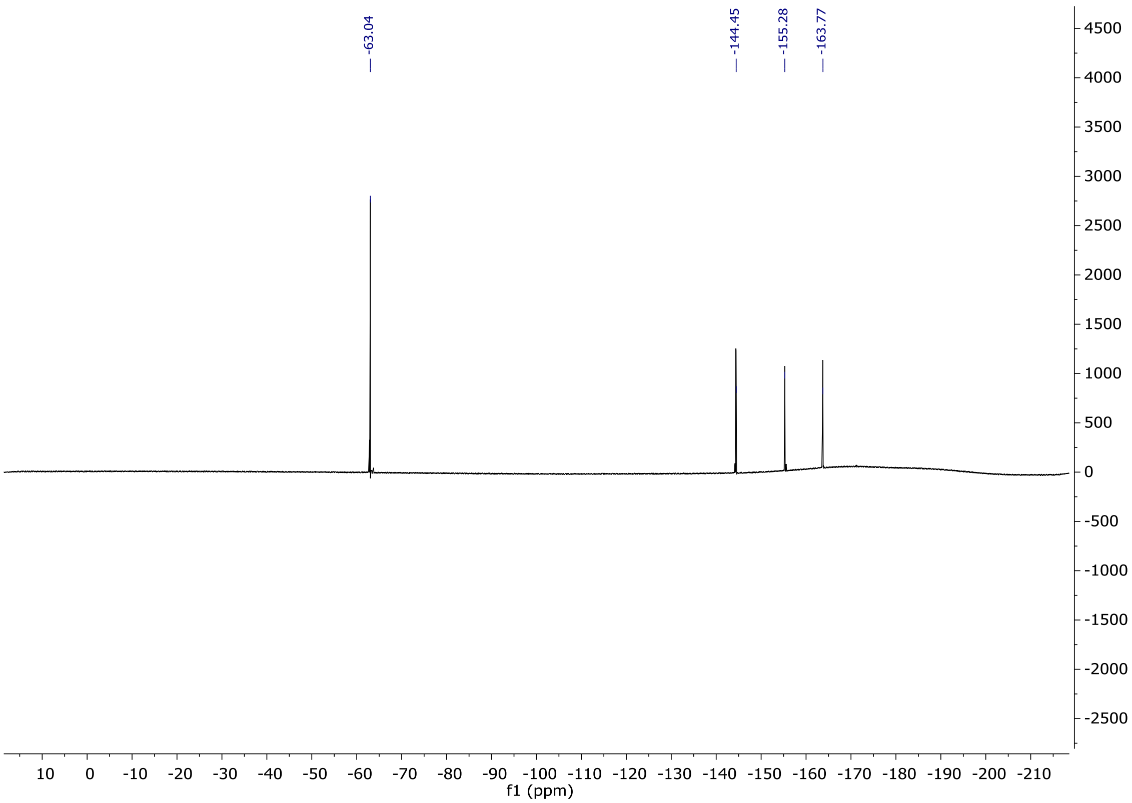

Supplementary Fig. 35. Quantification of the detachment of CF_3Bn-ON moiety from CF_3Bn-ON-CNF film. A representative ^{19}F NMR spectrum showing signals of the standard and detached moiety.

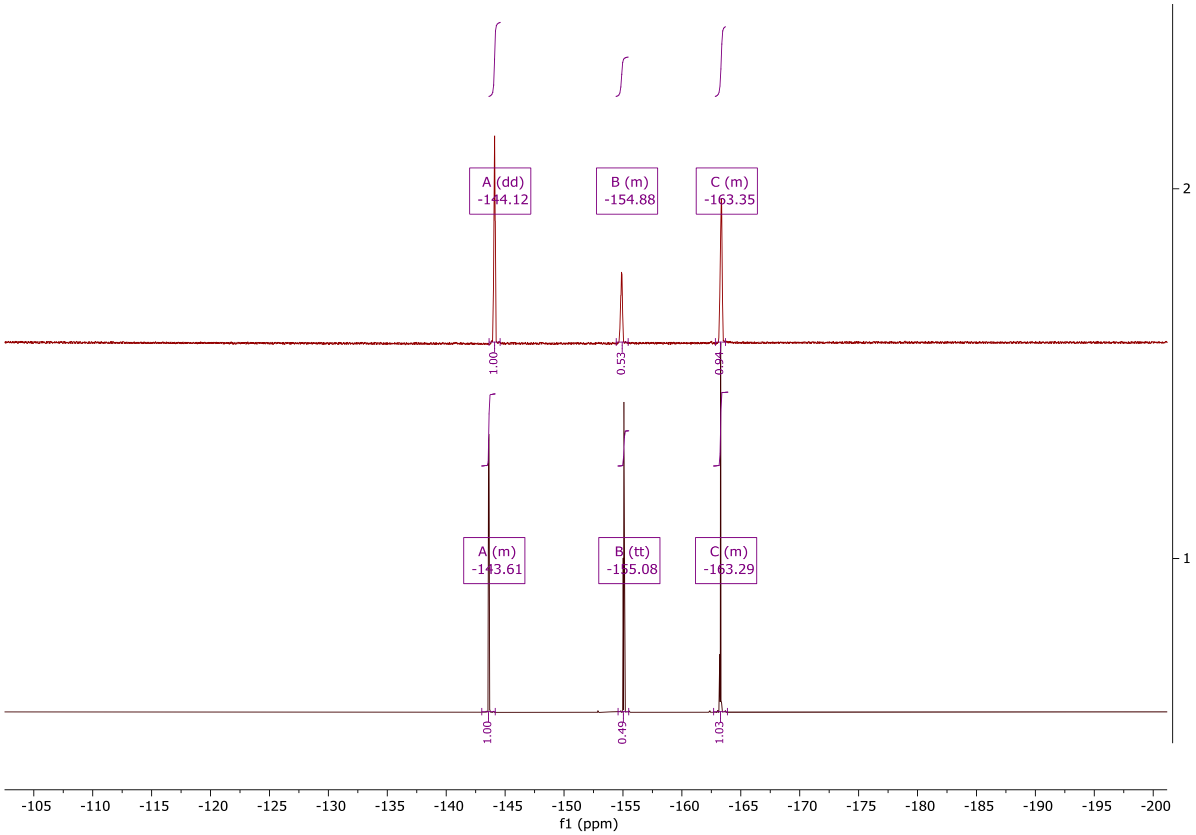

Supplementary Fig. 36. Evaluation of the stability of the standard (pentafluoro benzyl bromide) under reaction conditions. Top: ^{19}F NMR spectrum of pentafluoro benzyl bromide treated in 1M(HCl) acetone/water mixture for 96 hours at room temperature. Bottom: ^{19}F NMR spectrum pentafluoro benzyl bromide.

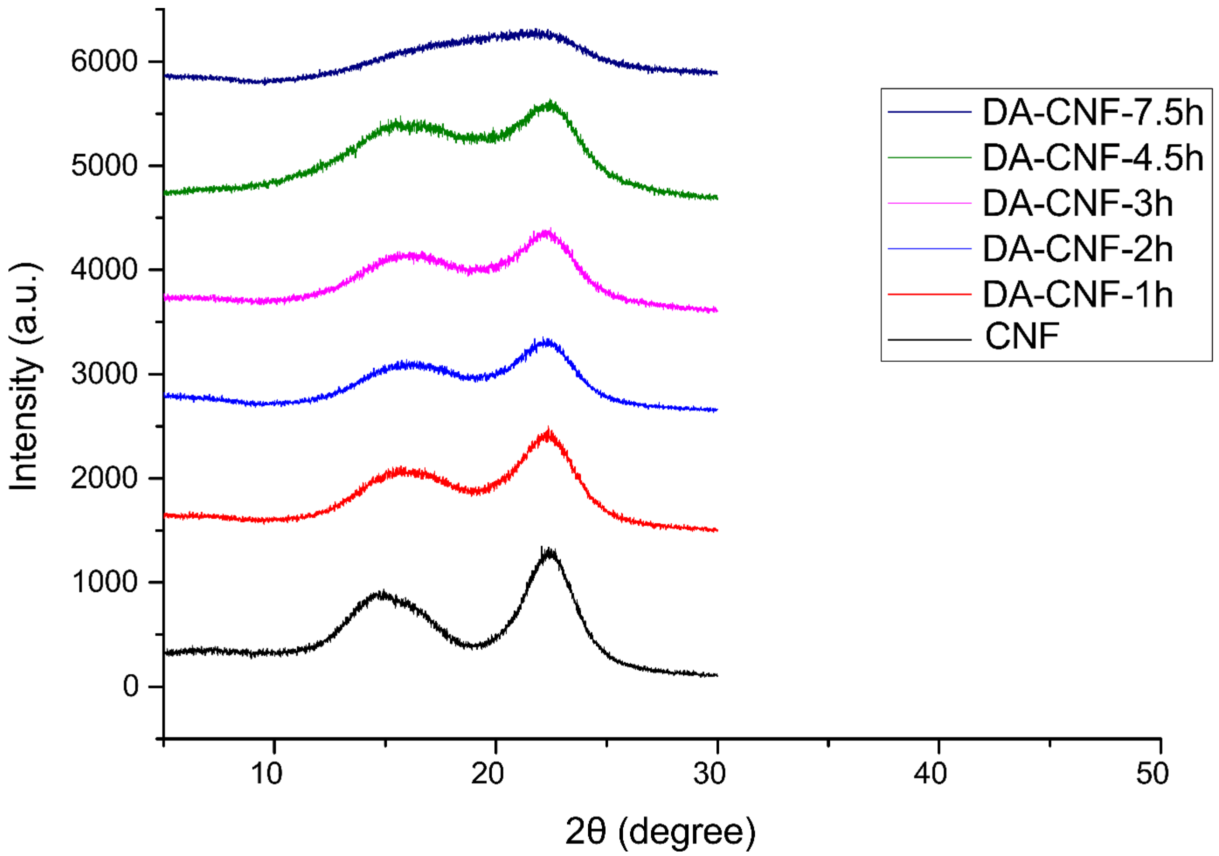

Supplementary Fig. 37. XRD spectra of CNF and DA-CNF obtained by periodate oxidation of CNF for 1 – 7.5 hours

Supplementary Table 1. Oxidation of CNF wet cakes to DA-CNF

Entry	$NaIO_4$, g	Reaction time, h	Aldehyde, mmol/g	Shrinkage, %
1	2.2	3	3.2	24
2	1.1	7.5	3.4	26
3	0.3	38	3.5	25
4	2.2	1	1.5	1
5	2.2	2	2.6	6
6	2.2	4.5	4.8	47
7	2.2	7.5	5.5	54
8	2.2	24	7.7	62

The reactions were performed in 40 mL of water.

Supplementary Table 2. Mw of DA-CNFs

Entry	CNF	Aldehyde, mmol/g	Mw (AGU_{DA-CNF}), g/mol
1	DA-CNF-2h	2.6	161.6
2	DA-CNF-3h	3.2	161.5
3	DA-CNF-4.5h	4.8	161.2

Supplementary Table 3. Summary of optical properties of holo-CNF, DA-CNF and selected R-DA-CNF

Entry	Sample	Transmittance (600 nm), %	Transmittance (300 nm), %	Haze (600 nm), %	Thickness, mm
1	DA-CNF (smooth)	89	57	9	0.055
	DA-CNF (rough)	87	46	50	0.055
2	Bn-ON-CNF (smooth)	88	17	22	0.150
3	Bn-ON-CNF (rough)	88	17	60	0.140
4	Allyl-ON-CNF (smooth)	89	8	24	0.200
5	PFB-ON-CNF (smooth)	88	12	31	0.160
6	Me-ON-CNF (rough)	86	35	80	0.070
7	Me-ON-CNF (smooth)	89	34	11	0.070
8	Holo-CNF (smooth)	81	-	50	0.033
8	Holo-CNF (smooth)	58	3	-	0.125

Supplementary References

1. S. Cui, H. Jiang, L. Chen, J. Xu, W. Sun, H. Sun, Z. Xie, Y. Xu, F. Yang, W. Liu, F. Feng, W. Qu, Design, synthesis and evaluation of wound healing activity for β-sitosterols derivatives as potent Na+/K+-ATPase inhibitors. *Bioorg. Chem.* **98**, 103150 (2020).
2. M. Austin, O. J. Egan, R. Tully, A. C. Pratt, Quinoline synthesis: scope and regiochemistry of photocyclisation of substituted benzylidenecyclopentanone O-alkyl and O-acetyloximes. *Organic & Biomolecular Chemistry* **5**, 3778-3786 (2007).
3. C. Premi, A. Dixit, N. Jain, Palladium-Catalyzed Regioselective Decarboxylative Alkylation of Arenes and Heteroarenes with Aliphatic Carboxylic Acids. *Org. Lett.* **17**, 2598-2601 (2015).
4. L. Agnetta, M. Bermudez, F. Riefolo, C. Matera, E. Claro, R. Messerer, T. Littmann, G. Wolber, U. Holzgrabe, M. Decker, Fluorination of Photoswitchable Muscarinic Agonists Tunes Receptor Pharmacology and Photochromic Properties. *J. Med. Chem.* **62**, 3009-3020 (2019).
